# Structured tracking of alcohol reinforcement (STAR) for basic and translational alcohol research

**DOI:** 10.1038/s41380-023-01994-4

**Published:** 2023-02-27

**Authors:** Alex R. Brown, Hannah E. Branthwaite, Zahra Z. Farahbakhsh, Snigdha Mukerjee, Patrick R. Melugin, Keaton Song, Habiba Noamany, Cody A. Siciliano

**Affiliations:** 1 Department of Pharmacology, Vanderbilt Brain Institute, Vanderbilt Center for Addiction Research, Vanderbilt University, Nashville, TN 37232 USA; 2 Department of Neurobiology, Harvard Medical School, Boston, MA USA

**Keywords:** Neuroscience, Physiology

## Abstract

There is inherent tension between methodologies developed to address basic research questions in model species and those intended for preclinical to clinical translation: basic investigations require flexibility of experimental design as hypotheses are rapidly tested and revised, whereas preclinical models emphasize standardized protocols and specific outcome measures. This dichotomy is particularly relevant in alcohol research, which spans a diverse range of basic sciences in addition to intensive efforts towards understanding the pathophysiology of alcohol use disorder (AUD). To advance these goals there is a great need for approaches that facilitate synergy across basic and translational areas of nonhuman alcohol research. In male and female mice, we establish a modular alcohol reinforcement paradigm: Structured Tracking of Alcohol Reinforcement (STAR). STAR provides a robust platform for quantitative assessment of AUD-relevant behavioral domains within a flexible framework that allows direct crosstalk between translational and mechanistically oriented studies. To achieve cross-study integration, despite disparate task parameters, a straightforward multivariate phenotyping analysis is used to classify subjects based on propensity for heightened alcohol consumption and insensitivity to punishment. Combining STAR with extant preclinical alcohol models, we delineate longitudinal phenotype dynamics and reveal putative neuro-biomarkers of heightened alcohol use vulnerability via neurochemical profiling of cortical and brainstem tissues. Together, STAR allows quantification of time-resolved biobehavioral processes essential for basic research questions simultaneous with longitudinal phenotyping of clinically relevant outcomes, thereby providing a framework to facilitate cohesion and translation in alcohol research.

## Introduction

Alcohol (ethanol) is among the most studied chemical compounds in history [1] and continues to draw great interest from basic researchers across a wide range of disciplines including structural chemistry [2], pharmacology [3], toxicology [4], physiology [5], evolutionary biology [6], neuroscience [7], and reinforcement learning [8]. Alcohol is widely consumed for its psychoactive properties [9] and although severe adverse consequences only occur in a subset of drinkers [10], alcohol use remains an ongoing global health crisis linked to more than 5% of all premature deaths worldwide [11]. As such, there are long-standing efforts in translational and clinical research aimed at understanding the biological consequences of alcohol consumption to develop therapeutic interventions for alcohol use disorder (AUD) [12, 13]. However, there is a lack of methodologies that allow researchers across disciplines to investigate alcohol in a common framework, which likely represents a missed opportunity to facilitate advances in both basic and translational endpoints.

Integration across the highly diverse subfields of alcohol research in nonhuman subjects is needed to maximize the insight and advances gained [14, 15]. Indeed, retrospective evaluations of animal disease models indicate the utility of frameworks which explicitly create avenues for researchers to synthesize findings and provide continuity across subfields [16–21]. In the absence of purposeful development of methodological solutions, there is a natural tendency for fields to split along basic and translational lines as highly specialized paradigms are pushed towards partially divergent goals: basic investigations require flexibility of experimental design as hypotheses are rapidly tested and revised, while animal models intended for preclinical to clinical translation emphasize standardized protocols to allow comparison of specific outcome measures [22, 23]. The tendency for basic experimental studies and preclinical testing to use non-overlapping methodologies, without explicit efforts to integrate conclusions, impedes progress and translation [18, 24–26]. Accordingly, utilizing flexible procedures that allow for experimenter-specific modifications while retaining conceptual consistency, as opposed to rigid protocols, is likely to catalyze breakthrough discoveries [16, 27–30].

Here, guided by meta-analytic assessments of successes and shortcomings in preclinical disease research, we sought to design a cohesive model for bridging basic and translational investigations into the biobehavioral activity of alcohol. In male and female mice, we establish the Structured Tracking of Alcohol Reinforcement (STAR) framework, which provides a schema for addressing basic questions regarding alcohol reinforcement and consumption simultaneous with assessment of multivariate drinking outcomes and experience-dependent phenotype dynamics. The STAR framework utilizes operant alcohol self-administration which, by modifying schedules of reinforcement, allows experimenters to interrogate a wide array of processes [31, 32]. The unifying element of STAR is assessment of two outcome measures with predictive and construct validity within a reinforcement framework: alcohol intake and continued drinking despite punishment. In place of a rigid protocol, STAR implements a multivariate phenotyping analysis to categorize subjects based on relative variance in alcohol intake and punishment sensitivity. By describing expression of the wide individual differences engendered by these behaviors using group normalized values, robust phenotypes are consistently captured over a range of experimental scenarios. STAR is amenable to the procedural flexibility required to pursue basic research questions, provides high-resolution timeseries readouts of appetitive and consummatory behavior, and is optimized to allow seamless interfacing with neurotechnologies for real-time interrogation of the biological underpinnings of alcohol reinforcement. Critically, the STAR framework is explicitly designed to allow integration, rather than competition, with the diverse extant models that have been developed in the preclinical alcohol field [13, 33–35]. These features are intended to facilitate interdisciplinary communication and advance translational value with minimal constraints on experimental design.

To this end, we establish and parameterize the STAR framework, demonstrate integration with popular alcohol exposure methods, provide a publicly accessible protocol repository, and utilize coupled liquid chromatography-mass spectrometry (LC-MS) analysis of cortical and brainstem tissues to reveal the neurochemical signatures associated with heightened AUD-relevant drinking behaviors. Together, we establish a novel framework intended to facilitate accumulation of knowledge gained via disparate methodologies across alcohol subfields while retaining cross-study comparability using a standardized process for multivariate phenotype classification.

## Results

### Individual differences in alcohol consumption and punishment-sensitivity emerge over time and experience in male mice

To inform the design of the STAR framework, we capitalized on the recent efforts in several fields to identify features of preclinical models that improve the likelihood of translation from animals to humans. Consistent themes in the literature include the need for (1) quantitative outcome measures of specific disease subdomains (rather than attempting to model the disorder in entirety), (2) utilization of sample heterogeneity to define disease-relevant phenotypes via methodologies that can be readily understood by basic researchers as well as clinicians, and (3) increased emphasis on behavioral endpoints that have conserved biological underpinnings across animal and human subjects [13, 21, 24, 25, 28, 29, 36–39]. Alcohol intake and compulsive drinking (operationally defined here as the degree to which intake is modulated by punishment) are both central to AUD symptomatology, and are observable variables which can be readily quantified in animals [40–42]. We recently identified activity patterns in a population of neurons projecting from the prefrontal cortex to the periaqueductal gray area as a putative neuro-biomarker differentiating low alcohol intake, high alcohol intake, and compulsive drinking phenotypes in mice [43]. Subsequently, these findings were explicitly evaluated in two separate studies in humans which both found highly congruent relationships between cortical-brainstem activity and alcohol use vulnerability [44, 45]. Together, this provides strong support for the idea that a quantitative framework centered around individual differences intersectionally defined by alcohol intake and compulsive drinking may represent an opportunity to fulfill the three recommendations above. Here, we formalize a framework for quantifying these domains within a flexible reinforcement learning task and provide publicly available protocols and resources which together allow this approach to be readily transferred across laboratories and experimental questions.

**Fig. 1:**
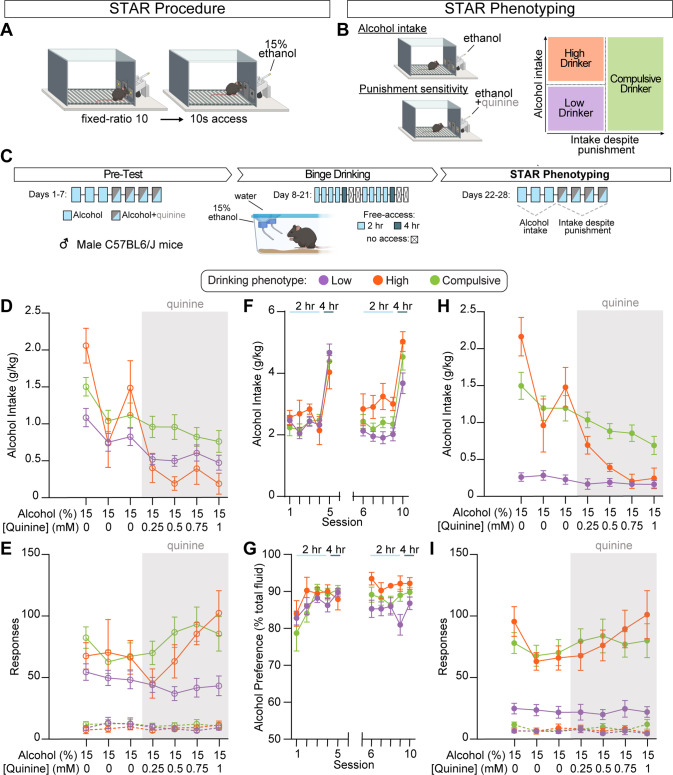
Structured tracking of alcohol reinforcement before and after binge drinking. **A** The primary methodology of the STAR framework is assessment of alcohol consumption during an alcohol reinforcement task wherein responding is reinforced under a fixed-ratio 10 schedule of reinforcement by extension of the alcohol sipper for 10 s. **B** Findings using diverse alcohol reinforcement procedures are integrated within a common conceptual framework using a straightforward phenotyping analysis to categorize subjects based on levels of alcohol intake and continued drinking despite punishment. **C** Experimental timeline. Here we utilized the STAR framework to examine the emergence of individual differences over time and drinking experience. All subjects are tested under identical experimental conditions and group assignments are assigned based on phenotyping from the post-test data. Alcohol intake (**D**) and operant responding (**E**) across three alcohol self-administration sessions. Graded concentrations of quinine are then added to the alcohol solution across sessions to test the sensitivity of alcohol reinforcement to punishment. Subsequently, animals are allowed free access to alcohol during a two-bottle choice procedure, which engenders high levels of alcohol consumption (**F**) and alcohol preference compared to water (**G**). During STAR phenotyping sessions, there were wide individual differences in alcohol intake with and without quinine punishment (**H**) as well as in operant responding for alcohol access (**I**). Low Drinkers, *n* = 19; High Drinkers, *n* = 5; Compulsive Drinkers, *n* = 17. Error bars indicate SEM.

The *sine qua non* components of the STAR framework are operant alcohol self-administration, where effort is required to gain access to alcohol (Fig. 1A), and a simple, standardized method of multivariate phenotyping where subjects are classified based on relative variance in alcohol drinking and drinking despite punishment (i.e. compulsive drinking) (Fig. 1B). Based on alcohol intake during punished and unpunished alcohol self-administration sessions, subjects are divided into one of three phenotypes: (1) “Low Drinkers” display below average intake both with and without punishment, (2) “High Drinkers” exhibit above average consumption of alcohol alone, but below average intake when punished, and (3) “Compulsive Drinkers” display above average alcohol intake despite punishment (Fig. 1B).

Here, we implement the STAR framework to investigate longitudinal dynamics in the expression of phenotypic drinking behaviors, and the degree to which phenotypes defined by alcohol intake and compulsive drinking may map onto other relevant domains. Importantly, the parameters described throughout were selected to pursue these specific questions, and the rationale for this design is detailed throughout. We have provided detailed protocols and behavioral code to allow ease of implementation (see “Methods”), but parameters should be altered as needed to best pursue the question at hand, provided that the definitions and calculation of the phenotyping analysis itself is consistent.

We first sought to determine whether phenotypic differences in alcohol intake can be observed during initial exposure to alcohol and assess the trajectory of these behaviors as it relates to the first opportunity to drink to intoxication in male mice. To assess drinking behaviors in a reinforcement learning paradigm, animals must first learn an operant contingency and for clear interpretation of behaviors at this timepoint it is necessary to dissociate operant learning from the motivational properties of alcohol [46]. Typically, acquisition of alcohol self-administration in rodent models is facilitated via addition of sweetening agents [47] or prior non-contingent alcohol exposure [48–51], either of which would obscure clear assessment of reinforcement and drinking behaviors throughout the initiation of alcohol use. To address this, we developed a protocol for controlled operant training in which mice are conditioned to respond to gain access to alcohol without the requirement of sweeteners or prior exposure, and opportunity for alcohol intake is limited until operant learning criteria are met. To control consumption, throughout all acquisition sessions alcohol intake was capped whereby sessions were terminated if 100 licks were registered. In a standard operant conditioning chamber, animals were first trained to lick for alcohol from a sipper tube containing alcohol (ethanol in water, 15% v/v) which remained extended for one-hour or until 100 licks were made, whichever came first. Subsequently, to gain access to the alcohol sipper animals were required to respond on a nose-poke under different reinforcement schedules (Fig. S1). Responding was initially reinforced under a fixed-ratio 1 (FR 1) schedule whereby a single response on the active nose-poke resulted in a 30 s extension of the sipper tube (Fig. S1A–D). Using phased criteria (see “Methods”), the response requirement was increased, and access period decreased across sessions until animals showed clear discrimination of the active vs inactive nose-poke under an FR 5 schedule for 10 s of alcohol access (Fig. S1E–L).

This protocol produces acquisition of operant alcohol self-administration with low attrition rates (87% acquired, Fig. S2A, B). Further, by imposing a fixed limit on intake per session, investigation of initial alcohol self-administration behavior can be performed independent of conflating variables related to differential learning rate, which can drive increased self-administration independent of propensity to drink per se, and limits variance in prior lifetime alcohol exposure across subjects (Fig. S2C). Lick contacts registered on the alcohol sipper were assessed across many sessions, with and without imposing a maximum lick number, and were found to be highly correlated with volumetric assessment of consumed alcohol as well as resulting blood alcohol concentration, and therefore provides a reliable, time-resolved readout of alcohol consumption throughout the session (Fig. S3). Importantly, while this acquisition protocol is useful for parsing the experimental question at hand, self-administration behavior at first opportunity to drink, it is not a requisite feature of the STAR framework; as discussed throughout, the use of normalized phenotyping allows for experimental parameters to be manipulated as needed while retaining a common framework.

Once acquisition criteria were met, responding during all subsequent self-administration sessions was reinforced under a FR 10 schedule for 10 s access to the sipper (Fig. 1A). Animals were tested across three daily one-hour sessions and allowed to respond for and drink alcohol, without the lick cap constraint imposed during acquisition sessions, to assess individual differences in the propensity to obtain and consume alcohol during subjects’ first opportunity to drink to intoxication (Fig. 1D, E). Next, alcohol was adulterated with increasing concentrations of the bitter tastant quinine over sessions (0.25, 0.5, 0.75, 1 mM) to assess drinking in the face of punishment (i.e., compulsive drinking). This initial assessment of alcohol intake and sensitivity to quinine punishment is referred to throughout as the pre-binge phase.

In addition to consideration of multimodal behavioral domains that contribute to disease states such as AUD [52, 53], clinical research has increasingly highlighted the importance of quantifying longitudinal changes in the expression of these traits [54–56]. Preclinical models, where limitations of cross-sectional experimental designs can be easily circumvented, have unique utility for parsing the development of AUD-relevant behaviors longitudinally; [57, 58] however, there is a paucity of animal studies that quantify longitudinal phenotype dynamics of alcohol drinking behaviors [13, 59]. To this end, we next used the STAR framework to examine the evolution of drinking behaviors over time and experience. Following pre-binge assessment of self-administration behavior, mice were given free-access to alcohol in a two-bottle choice procedure, based on the Drinking in the Dark paradigm [60, 61] but with concurrent access to water, for 0, 2- or 4-h a day (Fig. 1C). Two-bottle choice, where animals are given access to a water bottle and an alcohol bottle, is one of the most widely implemented animal models of alcohol drinking [62]. This approach has many variants but in general open access protocols engender high levels of intake with relatively little individual variance [43, 63]. After two weeks of testing in the two-bottle choice procedure (Fig. 1F-G), animals were returned to the operant conditioning chamber for assessment of alcohol consumption and punishment-sensitive drinking under identical conditions as pre-binge self-administration sessions (Fig. 1H–I). These sessions were used to perform STAR phenotyping whereby each animal was assigned to the Low, High, or Compulsive Drinker groups, and group assignment was then applied to the entire data set.

**Fig. 2:**
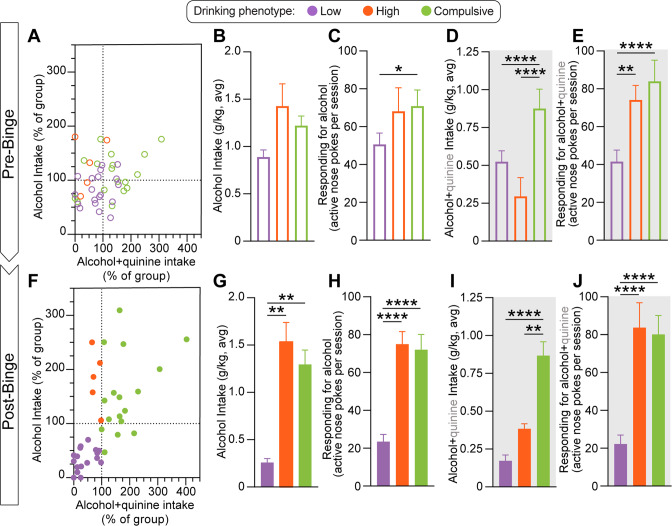
Phenotypic alcohol drinking behaviors emerge over time and experience. **A** Pre-binge: normalized distributions of alcohol intake (y-axis) and alcohol intake during quinine sessions (x-axis) from pre-test self-administration sessions. **B** Intake over the three pre-binge alcohol only self-administration sessions does not differ by phenotype (nested one-way ANOVA, F_(2, 6)_ = 1.691, *p* = 0.2615; 3 groups, 3 days per group, 122 total values). **C** Active nose-poke responses over the three pre-binge alcohol only self-administration sessions differs by phenotype with Compulsive Drinkers responding more than Low Drinkers (nested one-way ANOVA, F_(2, 119)_ = 4.667, *p* = 0.0112; 3 groups, 3 days per group, 122 total values). **D** Intake during pre-binge alcohol+quinine self-administration sessions differs by phenotype with greater intake in Compulsive Drinkers compared to Low or High Drinkers (nested one-way ANOVA, F_(2, 161)_ = 15.31, *p* < 0.0001; 3 groups, 4 days per group, 164 total values). **E** Active nose-poke responses during pre-binge alcohol+quinine self-administration sessions differs by phenotype, with Compulsive and High Drinkers displaying higher response rates compared to Low Drinkers (nested one-way ANOVA, F_(2, 161)_ = 20.32, *p* < 0.0001; 3 groups, 4 days per group, 164 total values). **F** Post-binge: normalized distributions of alcohol intake (y-axis) and alcohol intake during quinine sessions (x-axis) from post-binge self-administration sessions. **G** High and Compulsive Drinkers display greater alcohol intake over the three post-binge alcohol only self-administration sessions compared to Low Drinkers (nested one-way ANOVA, F_(2, 6)_ = 20.13, *p* = 0.0022; 3 groups, 3 days per group, 122 total values). **H** High and Compulsive Drinkers display higher active nose-poke responses over the three post-b alcohol only self-administration sessions compared to Low Drinkers (nested one-way ANOVA, F_(2, 120)_ = 44.15, *p* < 0.0001; 3 groups, 3 days per group, 123 total values). **I** Compulsive Drinkers have higher intake over the four post-binge alcohol+quinine self-administration sessions compared to High and Low Drinkers (nested one-way ANOVA, F_(2, 9)_ = 44.90, *p* < 0.0001; 3 groups, 4 days per group, 164 total values). **J** High and Compulsive Drinkers display higher active nose-poke responses over the four post-binge alcohol+quinine self-administration sessions compared to Low Drinkers (nested one-way ANOVA, F_(2, 161)_ = 50.92, *p* < 0.0001; 3 groups, 4 days per group, 164 total values). All post hoc comparisons used Tukey’s test: *p < 0.05; **p < 0.01; ***p < 0.001; ****p < 0.0001. Alcohol only days: 3 groups x 3 days (Low Drinkers, *n* = 19; High Drinkers, *n* = 5 Compulsive Drinkers, *n* = 17); alcohol+quinine days 3 groups x 4 days (Low Drinkers, *n* = 19; High Drinkers, *n* = 5 Compulsive Drinkers, *n* = 17). Error bars indicate SEM.

During pre-binge self-administration, there was limited variance across subjects (Fig. 2A). We found no differences between phenotypes in alcohol intake (Fig. 2B), but Compulsive Drinkers showed increased operant responding for sipper access (Fig. 2C) during pre-binge self-administration sessions for unadulterated alcohol. Phenotypes differed in self-administration when alcohol drinking was punished via quinine adulteration, with Compulsive Drinkers displaying higher intake than Low Drinkers and High Drinkers (Fig. 2D). Both Compulsive Drinkers and High Drinkers displayed higher rates of responding than Low Drinkers during the quinine adulteration sessions (Fig. 2E).

Phenotypes did not differ in total alcohol intake during the two-bottle choice procedure (Fig. S4). Nonetheless, wide individual differences in alcohol self-administration and sensitivity to punishment emerged following binge alcohol drinking (Fig. 2F). During unpunished STAR phenotyping self-administration sessions, High and Compulsive Drinkers both displayed greater alcohol consumption and operant responding compared to Low Drinkers (Fig. 2G, H). Although High and Compulsive Drinkers did not differ in self-administration behavior during alcohol only sessions, alcohol consumption in Compulsive Drinkers was much less sensitive to punishment compared to both Low and High Drinkers (Fig. 2I). Interestingly, the impact of punishment on operant responding was dissociable from intake and varied by phenotype as both High and Compulsive drinkers displayed high response rates throughout the punishment sessions despite widely differential consumption (Fig. 2I–J). Importantly, phenotypic behaviors were not associated with differences in subject weight (Fig. S5), nor were they due to differential sensitivity to quinine itself as there were no differences in quinine avoidance when presented in a taste preference assay outside of the context of alcohol (Fig. S6).

These results confirm that repeated drinking can reveal widely disparate latent behavioral traits, even when subjects are exposed to similar amounts of alcohol. Indeed, limited differences in drinking behavior observed during initial testing were exacerbated over time and experience to form widely disparate phenotypes, despite equivalent alcohol consumption during two-bottle choice testing. Furthermore, these data highlight that multivariate assessments reveal distinct phenotypes that would go undetected using unidimensional measures. For example, High Drinkers, which are defined by covariance across two traits, show heightened operant responding during punished STAR phenotyping sessions despite displaying low levels of consumption (Fig. 2G–J). The dissociable measures of intake and responding provides an additional axis of drinking-related behavior by which to evaluate subjects, and may allow discrete assessment of appetitive and consummatory behavioral processes which occur during alcohol use [64, 65].

### STAR provides quantification of granular behavioral patterns in tandem with longitudinal phenotype dynamics

The procedures and analyses presented thus far establish a high-throughput platform for quantitative assessment of multiple clinically-relevant outcome measures, determine emergence of phenotypic behaviors between subjects and across time and, by disseminating empirical findings within a straightforward conceptual framework, can be readily integrated with translational and clinical literatures. However, from a scientific and practical perspective, for the STAR framework to facilitate cohesion across subfields this approach must also offer features that are attractive to alcohol researchers with diverse experimental questions and technical approaches.

Integration of modern neurotechnologies with alcohol drinking procedures in rodents represents an ongoing challenge in the field due to the requirement of specific task structures, specialized hardware, and time-resolved behavioral readouts [23, 66–68]. STAR is explicitly designed and optimized to circumvent these challenges. Use of motorized sippers for alcohol delivery and temporally precise lick detection allows techniques that require tethering and head-mounted hardware, such as miniature microscopes or fiber photometry, to be implemented without alterations to the procedure [43]. Further, responses on the active and inactive operanda, reinforced responses, and consumption of alcohol are inherently spaced in time by virtue of using an FR 10 schedule of reinforcement, allowing clear comparisons of time-resolved neural measurements across multiple stimuli [23, 66]. Timeseries behavioral data is recorded throughout all self-administration sessions and provides a means for interfacing with in vivo recording and manipulation techniques as well as assessment of granular behaviors that occur during drinking such as microstructural patterns of licking behavior (Fig. S7).

**Fig. 3:**
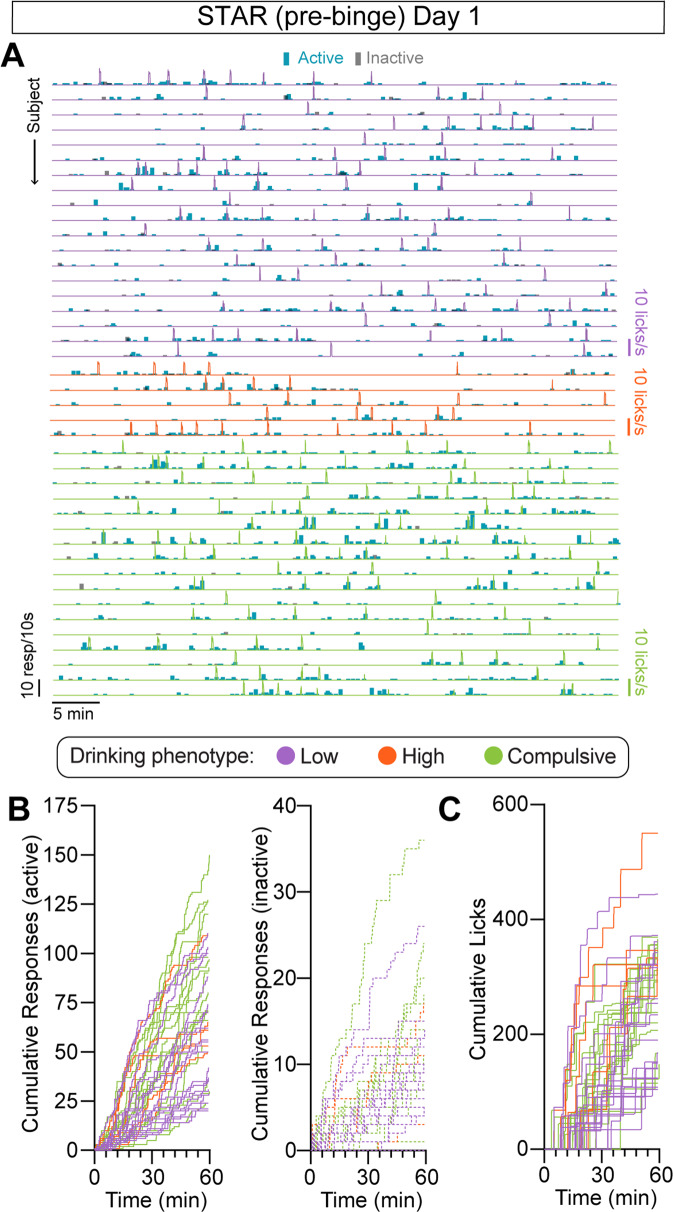
STAR procedure provides measurement of microstructural behavioral patterns for detailed behavioral analysis and seamless integration with in vivo neurotechnologies. Precise timing of behavioral events is recorded (sampled at 1 kHz, binning denoted by graph), allowing online interfacing with techniques for observing and manipulating neural activity or with *post hoc* analysis pipelines, without altering any of the task parameters. **A** Superimposed event records of response rate for active responses (blue, 10 s binning), inactive responses (gray, 10 s binning), and licks (colored by phenotype of subject, 1 s binning) for 15 subjects during the pre-binge self-administration session. Individual differences across subjects and phenotypes can be observed in temporal patterns of responding even when total responses over the course of the session display limited variability. **B** Cumulative records of active (left) and inactive (right) responses for individual subjects from the first pre-binge self-administration session. **C** Cumulative records of licks for individual subjects from the same session. Low Drinkers, *n* = 19; High Drinkers, *n* = 5; Compulsive Drinkers, *n* = 17.

While STAR phenotyping only requires volumetric measurement of alcohol intake, which is recorded as a single measurement by session, inclusion of timeseries measurements also provides a means for deep phenotyping. For example, cumulative records of operant responses can be generated to visualize within-session data (Fig. 3A, B), which can reveal additional phenotypic divergence that cannot be observed with gross behavioral readouts. Indeed, timeseries analysis of response and lick rate during subjects’ first opportunity to drink to intoxication illustrates wide between- and within-phenotype variance in temporal patterns of behavior (pre-binge day 1, Fig. 3C), even though there was little variance and no group differences detected in aggregate alcohol intake and responding at this timepoint (c.f. Fig. 2A–C).

### STAR outcome measures probe dissociable subdomains of drinking behavior and display putative reverse translation validity

Multivariate phenotyping is ostensibly useful, but it remains to be empirically tested if the specific outcome measures essential to STAR, intake of alcohol alone and intake in the face of punishment, provide insight in addition to a single measure. Alternatively, these measures may simply represent conceptually attractive redundancy. To begin to test these possibilities, we treated a subset of animals with the FDA-approved AUD pharmacotherapeutic naltrexone, which reduces alcohol intake in some AUD patients and reliably decreases alcohol intake in preclinical two-bottle choice testing [33, 69]. Following STAR phenotyping sessions, a subset of subjects was retested in a series of three alcohol self-administration sessions for alcohol alone or with punishment (0, 0.25, 0.5 mM quinine adulteration, ascending order). This series was tested twice, once with naltrexone treatment (1 mg/kg, i.p.) 30 minutes prior to the behavioral session and once with saline (random counterbalancing of treatment order across subjects).

We found that, in aggregate across all subjects tested, naltrexone treatment decreased alcohol intake during unpunished self-administration as compared to saline treatment (Fig. S8A). In contrast, though there was some reduction in intake during the quinine punishment sessions, these effects did not reach statistical significance in either punishment session (Fig. S8B, C). Furthermore, operant responding on the active nose-poke was not altered by naltrexone treatment during alcohol only or quinine punishment sessions (Fig. S8D–F). Dividing the data by subject phenotype revealed that naltrexone-induced reductions in unpunished alcohol intake were driven by Compulsive Drinkers while no effect of naltrexone was observed in Low Drinkers (Fig. S9A). Despite no effects of naltrexone on intake during punishment sessions or on active responding during any session type, Compulsive Drinkers displayed exceptionally wide variance between subjects (Fig. S9B–F). Together, these results are consistent with previous studies that alcohol intake during self-administration generally displays reverse translatability. Importantly, they also support the idea that the STAR framework provides additional information across dissociable axes of drinking-related behavior and that consideration of multifaceted within-subject measures provides a great deal of information that is not redundant with intake of alcohol alone.

### STAR phenotyping analysis captures variance across multiple behavioral domains

**Fig. 4:**
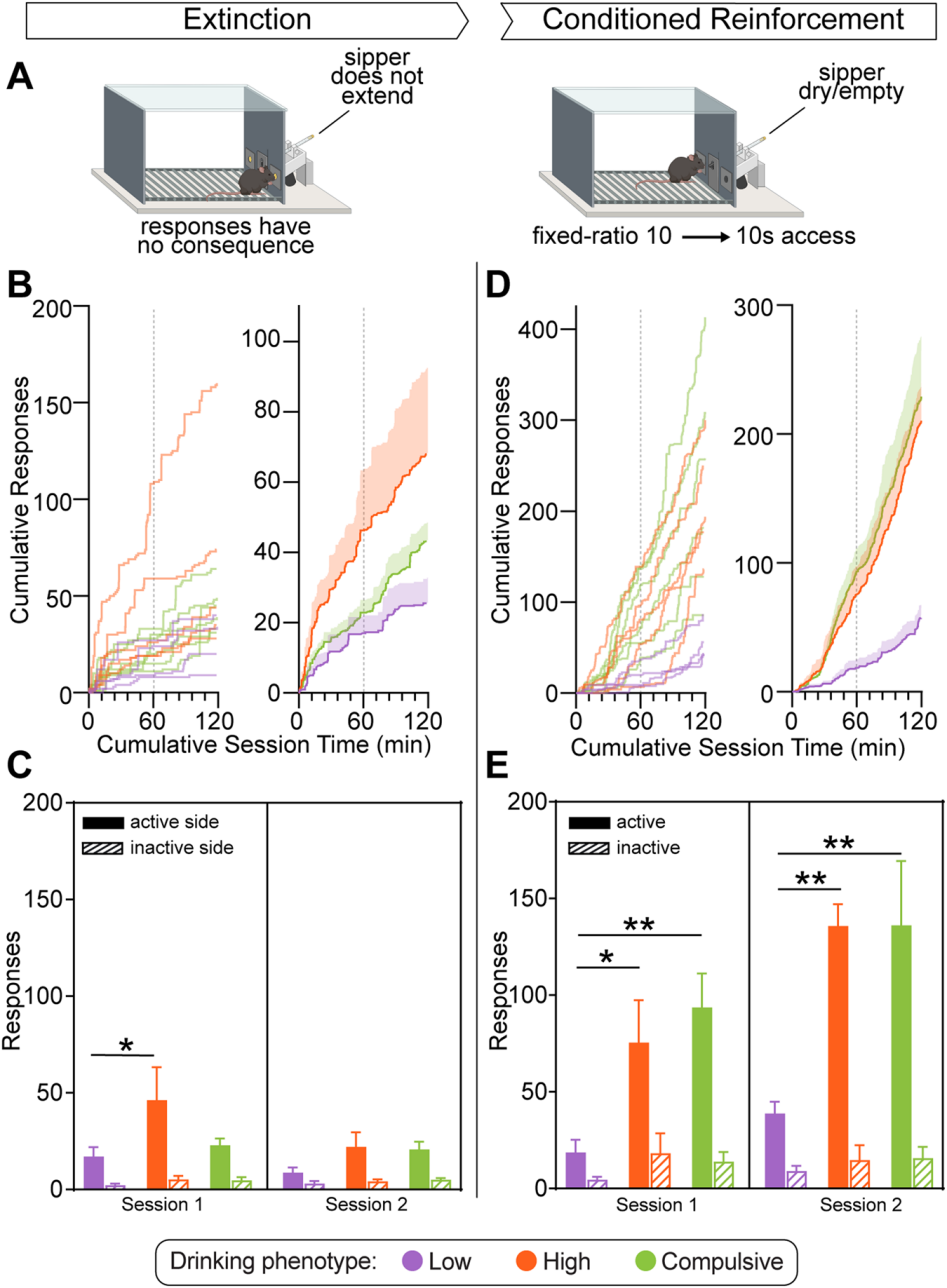
Differential responding for alcohol-conditioned stimuli across phenotypes. **A** Experimental timeline. Extinction: following completion of the STAR procedure and phenotyping (Fig. 1), animals are tested for responding under extinction conditions where responses on the previously active nose-poke have no consequence (Session 1–2). Next, animals are tested to determine conditioned reinforcement responding for alcohol-associated cues as a measure of alcohol seeking (Session 3–4). Conditioned Reinforcement (alcohol seeking): during conditioned reinforcement, the first response on the active nose-poke results in presentation of the sipper and responding during the remainder of both sessions is reinforced under a fixed-ratio 10 schedule of reinforcement. Following the first presentation, the contingency is identical to the pre-binge and STAR phenotyping self-administration sessions with the exception that the sipper does not contain any fluid, and thus the rate of responding is used as a measure of alcohol seeking in the absence of alcohol access. **B** Individual (left) and averaged (right) cumulative records of responses on the previously active nose-poke over extinction sessions (the two 60-minute sessions were concatenated into one record, denoted by the dotted line). **C** Responses on the previously active and inactive nose-pokes over the two extinction sessions. During the first extinction session, High Drinkers displayed greater responding on the previously active nose-poke compared to Low Drinkers (two-way ANOVA, nose-poke F_(1, 12)_ = 18.26, *p* = 0.0011; phenotype, F_(2, 12)_ = 2.067, *p* = 0.1693; nose-poke x phenotype F_(2, 12)_ = 2.040, *p* = 0.1727). During the second extinction session, a main effect of nose-poke remained but no differences were detected between phenotypes (two-way ANOVA, nose-poke F_(1, 12)_ = 19.08, *p* = 0.0009; phenotype, F_(2, 12)_ = 1.695, *p* = 0.2248; nose-poke x phenotype F_(2, 12)_ = 1.377, *p* = 0.2895). **D** Individual (left) and averaged (right) cumulative records of responses on the active nose-poke over conditioned reinforcement sessions (the two 60-min sessions were concatenated into one record, denoted by the dotted line). **E** Responses on the active and inactive nose-pokes over the two conditioned reinforcement sessions. High and Compulsive Drinkers responded more on the active nose-poke compared to Low Drinkers during both the first (two-way ANOVA, nose-poke F_(1, 12)_ = 29.24, *p* = 0.0002; phenotype, F_(2, 12)_ = 3.644, *p* = 0.0580; nose-poke x phenotype F_(2, 12)_ = 4.118, *p* = 0.0435) and second (two-way ANOVA, nose-poke F_(1, 12)_ = 45.97, *p* < 0.0001; phenotype, F_(2, 12)_ = 4.080, *p* = 0.0445; nose-poke x phenotype F_(2, 12)_ = 4.674, *p* = 0.0315) sessions. All comparisons used Tukey’s test: *p < 0.05; **p < 0.01. Low Drinkers, *n* = 4; High Drinkers, *n* = 5; Compulsive Drinkers, *n* = 6. Error bars indicate SEM.

Next, we sought to determine whether STAR phenotyping captures meaningful variance across behavioral domains which are not explicit to the analysis itself. To explore this, we focused on the influence of alcohol-conditioned stimuli, which can trigger seeking behavior in the absence of alcohol, a process which is separate from ongoing consumption of alcohol and thought to be of critical importance in precipitating relapse events [65, 70]. Because STAR utilizes an operant procedure where animals learn the contextual and discrete cues associated with alcohol consumption, rates of operant extinction as well as the motivational strength of alcohol-conditioned cues can be readily tested as needed for specific experimental questions. Two weeks following the completion of STAR phenotyping sessions, animals were tested for extinction resistance in two daily sessions of an extinction procedure, followed by two sessions of conditioned reinforcement to test the motivational strength of alcohol-associated stimuli (Fig. 4A). During extinction sessions, responses had no consequence, and the sipper tube was never extended. In conditioned reinforcement sessions, the first response on the active side was reinforced after which the schedule returned to FR 10 for the remainder of the sessions. Responding is reinforced by 10 s extension of the sipper, however, the sipper is dry/empty and no alcohol can be obtained during the session, though the rest of the experimental parameters were identical to those during phenotyping sessions.

During extinction, High Drinkers responded more on the previously active operandum than Low Drinkers during the first extinction session but not the second extinction session (Fig. 4B, C), suggesting that the High Drinkers phenotype is associated with resistance to extinction of responding for alcohol. During conditioned reinforcement, animals showed robust responding to obtain access to the alcohol-associated stimulus, with some subjects responding several hundred times over the course of the two 60 minute sessions (Fig. 4D). Both High and Compulsive Drinkers displayed greater responding than Low Drinkers across both sessions (Fig. 4E), demonstrating that High and Compulsive Drinker phenotype membership was predictive of heightened alcohol-conditioned reinforcement behavior measured in the absence of any alcohol access. Together, these data further demonstrate the diverse research questions that can be addressed within the STAR framework and highlight the utility of the STAR phenotyping method for capturing meaningful phenotypes that predict variance in related behavioral domains.

### Empirical assessment of sample size sensitivity of STAR phenotyping

A key component of the STAR framework is its modularity, which rests on group normalization such that phenotyping is determined based on values normalized as a percent of the group mean derived from subjects run under identical experimental conditions. As with any analysis of individual differences, sample size is a major determinant of outcomes of the analysis, and group normalization is likely to further amplify the potential influence of subject number. Determining the stability of STAR phenotyping as a function of sample size is thus paramount to its utility as a common framework that can be implemented across laboratories. To this end, we implemented a permutated resampling test whereby samples of varying size were iteratively generated via random subsampling of the full dataset (sample sizes 2 through 41 subjects, 100 iterations each). Each subsample was analyzed independently such that the group normalization was redetermined each time and phenotype assignments derived from the subsample analysis were then compared to those made from the full dataset. Thus, by permutation testing, the empirical probability of changing phenotype assignment as a function of sample size was determined (Supplementary Video 1 and Fig. S10A). We demonstrate that at samples sizes ≥12 subjects, phenotype assignment becomes highly stable in our dataset (Fig. S10B). For best practices in implementing STAR phenotyping across laboratories, we recommend a default minimum sample size of at least 15 subjects, tested under identical experimental conditions, before STAR phenotyping is performed. This recommendation is intended as a starting point, after which exact statistical parameters can be tailored to the experimental question and observed variability.

### Variance in alcohol reinforcement is associated with distinct patterns of neurotransmitter biosynthesis and metabolism in the medial prefrontal cortex and dorsal periaqueductal gray area

As discussed above, a major recommendation from the meta-analytics literature on preclinical models is increased emphasis on behavioral endpoints that have conserved biological underpinnings across animal and human subjects. This is, of course, a broad goal which cannot be addressed in a singular experiment or be expected to have a singular binary solution. Nonetheless, moving forward towards this goal is paramount. Thus, we next sought to explore the potential neurobiological markers that might distinguish STAR phenotypes with an overall goal of providing a basis for continued evaluation of congruence with the factors distinguishing individual differences in drinking behaviors in humans. We have previously demonstrated that optogenetic manipulations of medial prefrontal cortex (mPFC) neurons which project to the dorsal periaqueductal gray area (dPAG) produces bi-directional modulation of compulsive drinking behaviors [43]. Subsequently, two independent neuroimaging studies in human subjects explicitly evaluated a priori hypotheses based on our conclusions and found highly congruent findings implicating cortical-brainstem activity as a neuro-biomarker of vulnerability to compulsive drinking behaviors [44, 45]. We therefore focused our efforts on mPFC and dPAG to further this cross-subfield investigation of the biological basis of AUD-relevant behaviors [43–45, 71].

**Fig. 5:**
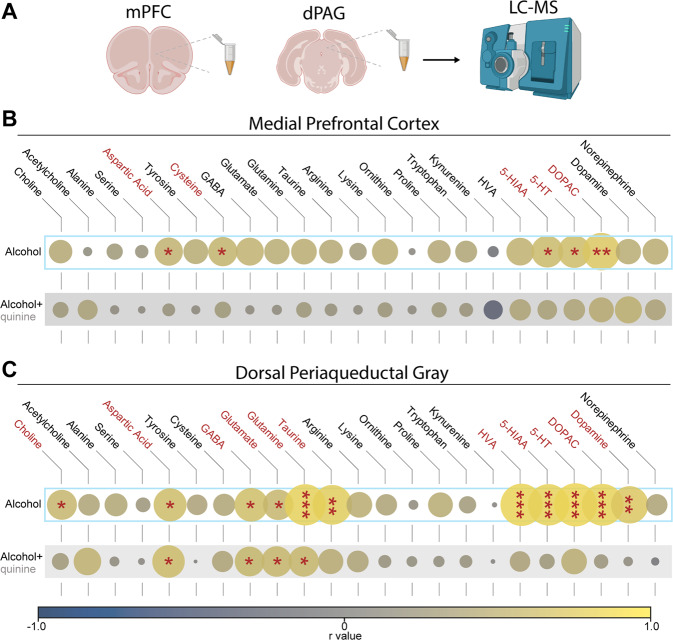
Alcohol use vulnerability is associated with augmented concentrations of biogenic amines in the medial prefrontal cortex and dorsal periaqueductal gray area. **A** Animals were sacrificed 24 h after a final alcohol self-administration session and samples of mPFC and dPAG were prepared for analysis using liquid chromatography-mass spectrometry (LC-MS). Twenty-three analytes were quantified in each region and correlations were performed to determine the relationship between analyte and self-administration during the post-test sessions for alcohol only and alcohol+quinine sessions. **B**, **C** r values for each correlation are indicated by circle size and color. Statistically significant correlations are marked with asterisks. Concentrations for each analyte by phenotype can be found in Supplementary Tables 1–3. **B** In mPFC, there was a positive correlation between aspartic acid, cysteine, 5-HIAAA, 5-HT, and DOPAC and alcohol self-administration during post-test sessions. No analytes correlated with intake during post-test alcohol+quinine sessions. **C** In dPAG, there were strong correlations between several analytes and alcohol only self-administration. Further, concentrations of aspartic acid, GABA, glutamate, and glutamine were positively correlated with alcohol+quinine intake. Spearman’s correlation coefficient: *p < 0.05; **p < 0.01; ***p < 0.001. Low Drinkers, *n* = 4; High Drinkers, *n* = 5; Compulsive Drinkers, *n* = 6.

To search for neuro-biomarkers that might explain variance in drinking behaviors across animals, we used coupled liquid chromatography-mass spectrometry (LC-MS) to perform neurochemical screening following the STAR procedure. Twenty-four hours after a final alcohol self-administration session, animals were sacrificed, samples of mPFC and dPAG were collected for analysis, and concentrations of 23 neurotransmitters, precursors, and metabolites were determined for each region across 15 subjects via LC-MS (Table S1**–**3). We first assessed potential markers of heightened alcohol intake or punishment-resistant alcohol intake by correlating concentrations of each analyte separately with the two STAR phenotyping metrics (normalized intake of alcohol or alcohol+quinine) (Fig. 5). In mPFC, we found that levels of 5-HT, its primary metabolite 5-HIAA, and the dopamine metabolite DOPAC were all positively correlated with intake of alcohol alone (Fig. 5B). We did not find significant relationships between mPFC levels of any of the 23 analytes and subjects’ normalized values of alcohol+quinine intake suggesting that mPFC may play a larger role in distinguishing a high drinking trait than compulsive drinking. In dPAG tissue, alcohol intake was associated with greater concentrations of 5-HT and dopamine and their metabolites as well as increased concentrations of GABA and glutamate (Fig. 5C). In contrast to mPFC, we found that concentrations of excitatory (glutamate, aspartic acid) and inhibitory (GABA) transmitters and their precursor (glutamine) were positively correlated with alcohol+quinine intake values (Fig. 5C, see Fig. S11 for additional data visualization). These results are congruent with those of Jia and colleagues [44] suggesting that excitatory/inhibitory balance in dPAG is a critical determinant of AUD vulnerability as well as phenotypes within AUD.

### STAR allows robust quantification of alcohol reinforcement and experience-dependent phenotype dynamics in female mice

It is critical for basic and translational research that studies examine both male and female subjects [72, 73]. Accordingly, female mice were tested in the STAR framework using the same methodology described for the male subjects above to determine if the same parameters provide robust assessment of individual differences in alcohol reinforcement in female mice as well. Using the same acquisition procedure, female subjects (Fig. S12) readily acquired operant responding for alcohol (92% met criteria, Fig. S13A). Similar to male subjects, phenotypes did not differ in days to acquisition or total alcohol intake throughout acquisition (Fig. S13C, D). Following acquisition, female subjects were tested under identical conditions to those described above, first assessing self-administration behavior at first opportunity to drink to intoxication and then performing STAR phenotyping after a two-week binge drinking protocol (Fig. S14).

Mirroring findings from male mice, in female mice we found no differences between phenotypes in alcohol intake (Fig. S15B), but Compulsive Drinkers showed increased operant responding for sipper access (Fig. S15C) during pre-binge self-administration sessions for unadulterated alcohol. Phenotypes differed in self-administration when alcohol was punished via quinine adulteration, with Compulsive Drinkers displaying higher intake than Low Drinkers (Fig. S15D). Both Compulsive Drinkers and High Drinkers displayed higher rates of responding than Low Drinkers during the quinine adulteration sessions (Fig. S15E).

In female mice, phenotypes did not differ in alcohol intake or preference versus water during the two-bottle choice procedure (Fig. S16). Nonetheless, as in male subjects, wide individual differences in alcohol self-administration and sensitivity to punishment were clearly evident during STAR phenotyping sessions performed following binge drinking (Fig. S15F). During STAR phenotyping self-administration sessions, High and Compulsive Drinkers both displayed greater alcohol consumption and operant responding compared to Low Drinkers (Fig. S15G, H). Although High and Compulsive Drinkers did not differ in self-administration behavior during alcohol only sessions, alcohol consumption in Compulsive Drinkers was much less sensitive to punishment compared to both Low and High Drinkers (Fig. S15I). Further, Compulsive Drinkers displayed higher operant responding than Low Drinkers during alcohol+quinine sessions (Fig. S15J). Importantly, as in males, phenotypic behaviors were not associated with differences in subject weight (Fig. S17) nor were they due to differential sensitivity to quinine itself, as there were no differences in quinine avoidance when presented in a taste preference assay outside of the context of alcohol (Fig. S18). Together, these results demonstrate that STAR allows robust testing in both male and female subjects. Detailed protocols, code, and related resources for implementing STAR have been made freely available (https://github.com/Siciliano-Lab/STAR).

## Discussion

Development of preclinical models and advancement towards clinical endpoints is a long-standing strength of the alcohol field. Decades of open debate as well as forward and reverse translation through diverse model species has steadily driven rigorous optimization of animal models, which ultimately produced multiple approved pharmacotherapeutic interventions for AUD [33, 35, 74, 75]. However, as clinical goals shift towards personalized medication strategies, there is increasing need for preclinical models of alcohol use that capture individual differences and incorporate multimodal assessments of specific behaviors within the AUD spectrum [52, 53]. At the same time, advances in neurotechnologies for interrogating in vivo cellular activity in real-time have rapidly transformed behavioral neurosciences but are often incompatible with established AUD models, resulting in a divergence of methodologies throughout the field [43, 76, 77]. Here, to address these issues, we establish an approach for Structured Tracking of Alcohol Reinforcement. We (1) validate that STAR phenotyping provides a readily transferable method for extracting meaningful classifications from longitudinal dynamics across multimodal behavioral domains, (2) demonstrate the modularity of STAR to flexibly pursue a range of experimental questions including incorporation of established preclinical models, and (3) identify multiple potential biomarkers of alcohol use vulnerability via neurochemical profiling of mPFC and dPAG.

Reinforcement learning is widely utilized and understood across disciplines, allowing for findings to be evaluated across paradigms and species, including human laboratory experiments [78, 79]. In the STAR framework, volitional ethanol consumption and punishment-resistant drinking are assessed in an operant reinforcement task. The measurement of alcohol intake and punishment resistance under operant reinforcement conditions and the subsequent phenotyping approach are the two core elements of STAR; any number of experimental questions can be flexibly addressed by incorporating additional tests within the operant framework (e.g., conditioned reinforcement) or combining with other assays (e.g., two-bottle choice). The phenotyping analysis itself is the only component that is entirely rigid—we recommend that the calculation is performed identically to allow comparison across studies (see methods and online repository).

STAR fulfills several criteria that have been identified across disciplines as advantageous features for preclinical models. We demonstrate that variance in alcohol intake and drinking despite punishment define three phenotypes, which we term Low, High, and Compulsive Drinkers. Importantly, this relatively simple approach captures variance across AUD-relevant outcome measures which were not included in the phenotyping analysis. For example, these phenotypes also diverge in operant response rates during alcohol self-administration, extinction resistance, and alcohol seeking. Furthermore, these measures appear to be readouts of dissociable processes within alcohol reinforcement which manifest differentially across phenotypes. As mentioned above, this structuring allows for the same framework to be used for a range of applications and specifically has the flexibility needed for basic science questions, with the formal framework that is helpful for preclinical translational pipelines.

The extinction and conditioned reinforcement assay, though not a requisite component of STAR, provides a robust readout of alcohol seeking behavior. Responding for drug-associated stimuli has been used widely in the stimulant literature as a measure of drug craving, but protocols for alcohol-conditioned responding are not as well established, particularly in mice. Interestingly, we see that most subjects display higher rates of responding in the second conditioned reinforcement session. We speculate that this effect may be analogous to the “incubation” phenomenon which has been extensively studied in the preclinical cocaine literature, whereby responding for drug-conditioned stimuli displays time-dependent increases over the course of abstinence [80, 81]. In this case, the increased responding appears to occur as a function of exposure to the alcohol-conditioned reinforcer, which may be more analogous to the clinical literature than a purely time-dependent phenomenon [82, 83]. Importantly, regardless of the underlying psychological drivers, this effect is driven by the presentation of the conditioned reinforcer, as responding under extinction conditions decreased over the course of two sessions.

We use this task to parse individual phenotypes that emerge over time and identify potential biomarkers for this phenotypic divergence. Using LC-MS to assess a range of analytes in mPFC and dPAG, we show that dopamine- and 5-HT-related analytes in both the mPFC and dPAG were positively correlated with intake during alcohol self-administration, but that there was no correlation between concentrations of any of the twenty-three molecules that were screened and alcohol intake during punished sessions. In contrast, excitatory and inhibitory transmitters in the dPAG were positively correlated with compulsive drinking. These data suggest that monoamine activity may be a widespread biomarker of high alcohol consumption, which is consistent with multiple clinical studies demonstrating elevated dopamine and 5-HT activity in heavy drinkers which can be observed across several brain regions and in circulating CSF. Furthermore, they add to mounting evidence that dPAG activity is a biomarker for vulnerability to compulsive drinking behaviors, and may represent a neurobiological substrate differentiating compulsive from high drinking phenotypes [43–45, 84].

Though we establish the use of STAR in both male and female mice in parallel experiments, we have not reported a direct analysis of the effect of sex on outcome measures as we feel that clear interpretation of any possible sexual dimorphisms requires additional experimentation, which are ongoing. Qualitatively, female mice displayed, in general, considerably lower alcohol intake than males during self-administration sessions (cf. Fig. 1 and Fig. S14) which invites the conclusion that males are therefore more prone to alcohol drinking than females. However, during two-bottle choice, in the same animals, these differences are not readily apparent (cf. Fig. S4A and S16A). This is reminiscent of recent findings in both the cocaine and opioid literatures showing that despite the long-held view that females have higher rates of self-administration, this relationship is abolished or even inverted when effort is required to obtain the drug or when presented as a discrete choice between reinforcers [85–87]. We and others have recently called for greater emphasis on sex x environment interactions, rather than claims of sexual dimorphism, when interpreting drug and alcohol self-administration data [88–90]. STAR provides a platform for investigations in female subjects going forward, as well as a quantitative framework for parsing sex x schedule x environment interactions when investigating sex differences specifically. The experiments presented were not designed to parse potential sex-specific effects and we caution against interpreting them as such. Rather, these parallel experiments were intended to establish the use of STAR for investigating both male and female subjects. The veracity of this approach is supported by the fact that the phenotypes can be clearly separated in both cases and that phenotype membership maps onto behavioral variables that are not included in the phenotype analysis itself (e.g., lever pressing) with a remarkable degree of similarity between the sexes.

Together, we establish a novel framework to address fundamental questions regarding the biological activity of alcohol and make this protocol freely available in hopes that it may facilitate cohesion across subfields to yield a greater understanding of AUD (see methods for resource repository). We propose STAR as a new flexible model for the study of the development of AUD and other SUDs with time and experience in both males and females. The ability to have a single task that tracks individual differences across the development of AUD is still of high relevance. STAR’s flexibility and ability to be easily integrated with existing technologies and tools makes it a strong model for studying individual development of increased alcohol use and compulsion from a behavioral, circuit, and population perspective.

## Materials and methods

### Animals

Male and female C57BL/6 J mice were used for all experiments (Jackson Laboratory). Animals arrived at 8 weeks of age and were allowed to acclimate to the facility for at least one week before any testing was performed. Animals were housed in groups of five under a reverse 12-h light-dark cycle with *ad libitum* water access. Chow (Picolab 5L0D, LabDiet) was given daily at slightly above caloric requirements such that a healthy adult weight was maintained throughout the course of experiments (males: 2.9–3 g/animal/day, females: 2.6–2.7 g/animal/day, corresponding to roughly 8.7 and 7.5 kcal^ME^/day for male and female subjects, respectively) [91, 92]. All experiments involving the use of animals were in accordance with NIH guidelines and approved by the Vanderbilt Institutional Animal Care and Use Committee.

### Structured tracking of alcohol reinforcement (STAR)

#### Overview & apparatus

All conditioning experiments were performed in an operant conditioning chamber (Skinner box, Med Associates). Two illuminated nose-poke ports were positioned on either side of a reward port with a retractable sipper tube (Med Associates, ENV-352AW). Nose pokes were detected by infrared beam breaks and licks were detected by a resistance lickometer (Med Associates, ENV-250C). All sessions were run in the dark during the animals’ dark cycle, and mice were continuously monitored via overhead infrared cameras (Security Camera Warehouse).

Subjects were tested once daily in one-hour sessions. 15% ethanol (v/v), prepared from 95% stock and diluted in ultrapure water, was used throughout unless otherwise noted. Mice were weighed at the conclusion of each daily session and fed after all animals completed their experiment for the day. Nose-pokes were illuminated to signal the start of each session (except for Magazine Training) and responding on the inactive nose-poke had no programmed consequence throughout (active side counter-balanced across animals).

#### Operant acquisition

During acquisition, sessions were terminated when the animal reached 100 licks or after one hour, whichever came first. Acquisition was performed in three phases, described below. If during any phase animals did not meet criteria for three consecutive sessions, they were returned to the previous phase until criteria for advancing were again met. If a subject was returned to a previous phase three times in total, they were removed from the experiment (see Figs. S2 and **S**12 for acquisition/attrition rates).

##### Magazine training

Animals were placed in the operant conditioning chamber with the sipper tube constantly extended. Once the 100-lick cap was reached, the sipper was retracted and the subject was moved onto operant conditioning acquisition the following day.

##### Operant conditioning

Criteria 1: Responding on the active nose-poke was reinforced by extension of the sipper for 30 s under an FR 1 schedule. Animals were moved onto the second criteria once the 100-lick cap was reached for two consecutive days.

Criteria 2: Responding continued to be reinforced under an FR 1 schedule, but the access period was shortened to 10 s. Animals were moved to the operant discrimination phase once the 100-lick cap was reached for two consecutive days.

##### Operant discrimination

Response requirement was raised to an FR 5 schedule for 10 s access. Animals were considered to have acquired once they displayed ≥ 70% responding rate on the “Active” side (Active/[Active+Inactive responses]) and reached the 100-lick cap for two consecutive sessions.

### STAR methodology before and after binge drinking

On the day following completion of acquisition, animals ran for a one-hour session per day (no lick cap) and sessions progressed in a fixed order regardless of performance. Responding was reinforced under an FR 10 schedule by presentation of the sipper for 10 s throughout.

#### Pre-binge

For days 1–3, the sipper contained 15% ethanol (v/v). For days 4–7, ethanol was adulterated with quinine at increasing concentrations for each session (250, 500, 750, and 1000 µM in 15% EtOH).

#### Binge

On the day following the completion of the pre-binge epoch, animals were given access to alcohol in a two-bottle choice procedure known to reliably result in binge levels of intake [43, 93]. Animals were individually placed into a clean home-cage and allowed 30 minutes to acclimate before two bottles containing either 15% ethanol (v/v) or water were placed on the cage top with the spout extending into the cage (side counterbalanced over days, bottles on at 2 hours into the dark cycle). For the first four days, animals were given two hours of access and on the fifth day, animals had four hours of access. The following two days were abstinence days in which the animals remained in their original home cages. This one-week cycle (four days of 2-hour access, one day of 4-hour access, and two days of abstinence) was repeated (14 days total).

#### STAR phenotyping

STAR phenotyping sessions started the day following the last day of abstinence of the binge epoch. The experiment was identical in structure and parameters to the pre-binge epoch.

To perform STAR phenotyping, two values are calculated for each animal and expressed as a percent of the sample mean: (1) subject’s average g/kg consumed over 3 alcohol only sessions [alcohol intake = (subject mean session 1–3 (g/kg)/mean of all subjects day 1–3 (g/kg))*100], and (2) subject’s average g/kg over 4 alcohol+quinine sessions [alcohol+quinine intake = (subject mean session 4–7 (g/kg)/ mean of all subjects day 4–7 (g/kg))*100]. Based on the calculated values for alcohol intake and alcohol+quinine intake animals are assigned to one of three phenotypes. Animals with below average values for both alcohol and alcohol+quinine are deemed “Low Drinkers” [alcohol <100% & alcohol+quinine <100%]; those with values above the average alcohol intake but below average alcohol+quinine were deemed “High Drinkers” [alcohol >100% & alcohol+quinine <100%]; animals with above average alcohol+quinine values were deemed ‘Compulsive Drinkers’ [alcohol+quinine >100%]. For visualization, we recommend plotting the values for all animals as a scatter plot where x = [alcohol+quinine values] and y = [alcohol only values].

### Extinction and conditioned reinforcement

During extinction, sipper tubes were filled with alcohol and placed in the extendable sipper identical to previous sessions, but nose pokes were not reinforced on either side and the sipper remained retracted throughout. Animals were tested under these conditions for two consecutive daily one-hour sessions, followed by two days of conditioned reinforcement testing. During conditioned reinforcement, sipper tubes were left empty/dry, but placed on the retractable port as usual. The first nose poke on the active side resulted in presentation of the dry sipper for 10 s. After the first presentation, the response requirement for 10 s access to the dry sipper was raised to FR 10 for the remainder of the session.

### Liquid chromatography/mass spectrometry analysis of neurochemical profiles

After all experiments were complete, a subset of animals performed a final day of alcohol self-administration (FR 10 → 10 s sipper access, 1 h sessions). In total, 24 h after the final self-administration, animals were sacrificed, brains were snap-frozen, and later sectioned on a cryostat (Leica) into 200 micron slices. Tissue punches (500 micron diameter) were used to obtain samples of mPFC and dPAG from the slices, which were then stored at -80 °C until just prior to analysis. Briefly, tissue punches were homogenized, 23 analytes of interest were quantified simultaneously from each sample using LC/MS, and analyte concentrations were normalized to total protein content of the sample (see Supplementary methods for details).

### Statistics

Statistical analyses were performed using GraphPad Prism (V9). Comparisons across three or more variables were made using one-way ANOVAs or two-way ANOVAs (followed by Tukey’s test when planned comparisons were made or interactions were detected). *P* values < 0.05 were considered to be statistically significant.

## Protocol availability

Programs for controlling operant boxes as well as detailed standard operating procedures, designed to be accessible to researchers who may not have extensive experience with operant conditioning, have been made available: https://github.com/Siciliano-Lab/STAR.

## Supplementary information

Supplemental Methods and Figures

Supplemental Video 1

Supplemental Video 1 Legend
