# Supplementary material for: Structured tracking of alcohol reinforcement (STAR) for basic and translational alcohol research
Source: Mol Psychiatry. 2023 Feb 27;28(4):1585–98. doi: 10.1038/s41380-023-01994-4 (PMC10208967; doi:10.1038/s41380-023-01994-4)
Supplement: Supplementary file 1 — Supplemental Methods and Figures [file 41380_2023_1994_MOESM1_ESM.pdf]

# Supplementary Materials for

## Structured tracking of alcohol reinforcement (STAR) for basic and translational alcohol research

Alex R. Brown[1], Hannah E. Branthwaite[1], Zahra Z. Farahbakhsh[1], Snigdha Mukerjee[1], Patrick R. Melugin[1], Keaton Song[1], Habiba Noamany[2], Cody A. Siciliano[1*]

[1]Department of Pharmacology, Vanderbilt Brain Institute, Vanderbilt Center for Addiction Research, Vanderbilt University, Nashville, TN 37232, USA.

[2]Department of Neurobiology, Harvard Medical School, Boston, MA

*Corresponding Author Email: cody.siciliano@vanderbilt.edu

## Supplemental Materials and Methods

**Blood alcohol concentrations:** Blood alcohol concentration (BAC) was assessed to determine if dependent measures of alcohol intake during self-administration, licks and volumetric readings taken before and after the session, were reliable measures of alcohol ingestion. To avoid confounding the experimental data with stress exposure, bleeds were performed on subjects that had already completed experimental procedures. Immediately following completion of a one-hour alcohol self-administration session, animals were restrained, the submandibular vein was punctured with a lancet (5.5mm, Goldenrod), and blood was collected with a heparinized capillary tube (Kimble). Blood was transferred to an Eppendorf tube and allowed to coagulate at room temperature for 30 min followed by centrifugation at 2500g for 15 min at 4°C. Ethanol concentration in the supernatant/serum was assessed using a commercially available assay (Pointe Scientific). Samples were run in triplicate along with a standard curve of known ethanol concentrations and read on a plate reader (Glomax-Discover, Promega).

**Quinine preference test:** For quinine preference testing, animals were separated into individual homecages for the duration of the experiment. Animals were given continuous access to two bottles, one always contained water and the other contained varying concentrations of quinine, and preference/avoidance of each concentration was assessed at 24-hour time points. Bottles were weighed every 24 hours to determine fluid consumption and the location of the bottles were switched to counterbalance for any side preference. Every concentration was presented once in each location, for a total of 48 hours of access per concentration in the following order: 0, 25, 100, 250, and 500 µM. The difference in weight of the bottles over the 24 hours was used as to calculate the volume of each solution consumed, and values from each bottle location were averaged to derive a percent preference value at each concentration.

**Naltrexone treatment test:** To test reverse translation validity, a subset of male mice were retested following STAR phenotyping sessions to determine the ability of naltrexone treatment to alter alcohol drinking behaviors. Naltrexone hydrochloride (Tocris, CAS# 16676-29-2) was dissolved in injectable saline at a concentration of 0.1 mg/mL. Mice were treated with naltrexone (1 mg/kg) or saline in a volume of 10 mL/kg, i.p. 30 minutes prior to the onset of behavioral testing. Animals were tested under the identical parameters and schedule to those

described during the main STAR task but in three consecutive daily sessions with ethanol being adulterated with 250 or 500μM of quinine on days 2 and 3 respectively. Animals repeated this series of three days twice, once with naltrexone treatment each day and once with saline, for a total of six days (order was counterbalanced across subjects).

**Liquid chromatography/mass spectrometry extended methods:**

15 male animals that had completed the entirety of the STAR paradigm as well as Conditioned Reinforcement and Quinine Preference testing were used in this analysis. Following the completion of all experiments (six months after STAR phenotyping), animals were given one more alcohol self-administration session, with identical as used during STAR, and sacrificed the following day at a 24 hour abstinence timepoint.

Small molecule neurotransmitters were measured in the Vanderbilt University Neurochemistry Core (Director, Dr. Ginger Milne).

*Internal standard synthesis:* Stock solutions of each analyte of interest (5ng/μL each) were made in DI water and stored at -80$^{o}$C. To prepare internal standards, stock solutions were derivatized in a similar manner to samples using isotopically labeled benzoyl chloride ($^{13}C_6$-BZC) as follows: 200μL of the stock solution was mixed with 400μL each of 500mM $NaCO_3$ (aq) and 2% $^{13}C_6$-BZC in acetonitrile was added to the solution. After two minutes, the reaction was stopped by the addition of 400μL 20% acetonitrile in water containing 3% sulfuric acid. The solution was mixed well and stored in 10μL aliquots at -80$^{o}$C. One aliquot was diluted 100x with 20% acetonitrile in water containing 3% sulfuric acid to make the working internal standard solution used in the sample analysis.

*Tissue buffer:* Tissues were homogenized, using a tissue dismembrator, in 100-750μL of 0.1M TCA, which contained $10^{-2}$ M sodium acetate, $10^{-4}$ M EDTA, and 10.5% methanol (pH 3.8). 10μL of homogenate was used for protein quantification. Samples were spun in a microcentrifuge at 10,000g for 20 minutes at 4$^{o}$C. The supernatant was removed for LC/MS analysis.

*Benzoyl chloride derivatization and LC/MS analysis:* Analytes in tissue extract supernatant were quantified using liquid chromatography/mass spectrometry (LC/MS) following derivatization with benzoyl chloride (BZC). 5μL of supernatant was then mixed with 10μL each of 500mM $NaCO_3$ (aq) and 2% BZC in acetonitrile in an LC/MS vial. After two minutes, the reaction was stopped by the addition of 10μL internal standard solution.

LC was performed on a 2.1 x 100 mm, 1.6µm particle CORTECS Phenyl column (Waters Corporation, Milford, MA, USA) using a Waters Acquity UPLC. Mobile phase A was 0.1% aqueous formic acid and mobile phase B was acetonitrile with 0.1% formic acid. MS analysis was performed using a Waters Xevo TQ-XS triple quadrupole tandem mass spectrometer. The source temperature was 150$^{o}$C, and the desolvation temperature was 400$^{o}$C. The LC gradient is shown below.

**Table S1. HPLC gradient for neurotransmitter analysis.**

| Time | Flow rate | %A | %B | Gradient |
|---|---|---|---|---|
| 0.00 | 0.200 | 99 | 1 | -- |
| 0.10 | 0.200 | 93 | 7 | 6 |
| 0.50 | 0.200 | 85 | 15 | 6 |
| 14.0 | 0.200 | 45 | 55 | 6 |
| 14.5 | 0.200 | 30 | 70 | 6 |
| 18.0 | 0.200 | 1 | 99 | 6 |
| 19.0 | 0.200 | 1 | 99 | 6 |
| 19.1 | 0.200 | 99 | 1 | 6 |
| 22.0 | 0.200 | 99 | 1 | 6 |

*Protein assay:* Protein concentration was determined using the BCA Protein Assay Kit (Thermo Scientific, Waltham, MA USA) in a 96-well plate format. 10µL of tissue homogenate was mixed with 200µL of mixed BCA reagent per manufacturer instructions. The plate was incubated at 23$^{o}$C for two hours before absorbance was measured by the plate reader (POLARstar Omega), purchased from BMG LABTECH Company.

**Table S2. mPFC tissue content of analytes by phenotype.** Values represent mean ± SEM nanogram of detected analyte per milligram of total protein (ng/mg).

| Analyte | Low Drinker (n = 4) | High Drinker (n = 5) | Compulsive (n = 6) | All (N = 15) |
|---|---|---|---|---|
| Choline | 5.633 ± 1.14 | 6.924 ± 6.924 | 7.37 ± 2.266 | 6.758 ± 1.12 |
| Acetylcholine | 5.653 ± 0.6441 | 6.584 ± 6.584 | 7.712 ± 2.126 | 6.787 ± 1.109 |
| Alanine | 1036 ± 122.7 | 1773 ± 1773 | 1010 ± 68.13 | 1271 ± 179.3 |
| Serine | 802.3 ± 125.9 | 1769 ± 1769 | 959.4 ± 156.8 | 1187 ± 221.8 |
| Aspartic Acid | 5434 ± 606.3 | 7279 ± 7279 | 6555 ± 920.6 | 6497 ± 590.1 |
| Tyrosine | 94.59 ± 19.48 | 240.5 ± 240.5 | 144 ± 34 | 163 ± 28.19 |
| Cysteine | 44.48 ± 5.346 | 50.8 ± 50.8 | 100 ± 44.9 | 68.81 ± 18.62 |
| GABA | 2232 ± 277.5 | 2710 ± 2710 | 3323 ± 917.1 | 2828 ± 411.8 |
| Glutamate | 19653 ± 2141 | 25307 ± 25307 | 24808 ± 4274 | 23600 ± 2479 |
| Glutamine | 6870 ± 920.8 | 9184 ± 9184 | 9179 ± 1805 | 8565 ± 954.9 |
| Taurine | 16597 ± 1586 | 19911 ± 19911 | 21987 ± 4854 | 19858 ± 2298 |
| Arginine | 272.2 ± 55.18 | 746.8 ± 746.8 | 347.6 ± 59.67 | 460.6 ± 97.83 |
| Lysine | 267.2 ± 30.87 | 564.2 ± 564.2 | 364.3 ± 55.47 | 405.1 ± 60.32 |
| Ornithine | 308.9 ± 81.41 | 1145 ± 1145 | 383.6 ± 52.47 | 617.5 ± 164.2 |
| Proline | 222.9 ± 35.46 | 516.9 ± 516.9 | 305.4 ± 41.11 | 353.9 ± 56.98 |
| Tryptophan | 103.8 ± 14.74 | 242 ± 242 | 158 ± 41.38 | 171.6 ± 30.81 |
| Kynurenine | 32.9 ± 6.167 | 78.29 ± 78.29 | 23.24 ± 1.63 | 44.16 ± 11.37 |
| HVA | 5.015 ± 1.218 | 6.664 ± 6.664 | 8.768 ± 3.241 | 7.066 ± 1.446 |
| 5-HIAA | 4.373 ± 2.825 | 9.69 ± 9.69 | 20.7 ± 12.68 | 12.67 ± 5.238 |
| 5-HT | 9.683 ± 2.687 | 12.53 ± 12.53 | 15.26 ± 2.945 | 12.86 ± 1.512 |
| DOPAC | 0.8875 ± 0.0876 | 1.178 ± 1.178 | 2.613 ± 1.132 | 1.675 ± 0.4783 |
| Dopamine | 7.895 ± 2.091 | 9.862 ± 9.862 | 16.76 ± 6.018 | 12.1 ± 2.596 |
| Norepinephrine | 35.55 ± 6.689 | 35.29 ± 35.29 | 43.39 ± 6.438 | 38.6 ± 3.509 |

**Table S3. dPAG tissue content of analytes by phenotype.** Values represent mean ± SEM nanogram of detected analyte per milligram of total protein (ng/mg).

| Analyte | Low Drinker (n = 4) | High Drinker (n = 5) | Compulsive (n = 6) | All (N = 15) |
|---|---|---|---|---|
| Choline | 4.415 ± 0.2812 | 7.828 ± 2.167 | 7.905 ± 2.014 | 6.949 ± 1.095 |
| Acetylcholine | 4.539 ± 0.5686 | 7.773 ± 2.031 | 7.069 ± 0.6194 | 6.629 ± 0.7656 |
| Alanine | 622.3 ± 152.2 | 656.5 ± 190.4 | 829.3 ± 165.9 | 716.5 ± 96.6 |
| Serine | 627.5 ± 288 | 264 ± 92.89 | 988.9 ± 545 | 650.9 ± 234.2 |
| Aspartic Acid | 4762 ± 184.1 | 7443 ± 2133 | 8504 ± 1481 | 7152 ± 954.7 |
| Tyrosine | 106.4 ± 35.77 | 106.3 ± 25.24 | 146.5 ± 49.66 | 122.4 ± 22.67 |
| Cysteine | 48.53 ± 6.64 | 99.77 ± 46.26 | 116.8 ± 46.82 | 92.94 ± 23.97 |
| GABA | 4421 ± 214.3 | 7360 ± 2488 | 7333 ± 1104 | 6565 ± 941.1 |
| Glutamate | 10021 ± 576.9 | 17480 ± 5092 | 18782 ± 3407 | 16012 ± 2259 |
| Glutamine | 5022 ± 199.7 | 9431 ± 2645 | 8723 ± 1395 | 7972 ± 1086 |
| Taurine | 4614 ± 215.1 | 8579 ± 2284 | 9639 ± 1893 | 7946 ± 1146 |
| Arginine | 267.5 ± 64.28 | 357.6 ± 109.3 | 548.7 ± 149.3 | 410 ± 74.57 |
| Lysine | 342.6 ± 70.53 | 432.7 ± 124.3 | 508.9 ± 126.8 | 439.1 ± 66.12 |
| Ornithine | 573.7 ± 360.2 | 166.9 ± 29.58 | 549 ± 220.8 | 428.2 ± 130 |
| Proline | 185.9 ± 58.79 | 186 ± 49.54 | 310.8 ± 97.83 | 235.9 ± 45.45 |
| Tryptophan | 94.64 ± 22.99 | 112.2 ± 28.63 | 152.8 ± 49.54 | 123.8 ± 22.41 |
| Kynurenine | 31.11 ± 7.913 | 26.99 ± 6.105 | 29.96 ± 4.781 | 29.28 ± 3.255 |
| HVA | 1.637 ± 0.1393 | 5.21 ± 1.028 | 5.286 ± 1.659 | 4.288 ± 0.8231 |
| 5-HIAA | 7.808 ± 1.729 | 14.61 ± 6.775 | 19.38 ± 8.297 | 14.7 ± 3.988 |
| 5-HT | 15.9 ± 1.419 | 28.82 ± 8.027 | 27 ± 4.544 | 24.64 ± 3.351 |
| DOPAC | 0.7104 ± 0.087 | 2.175 ± 0.4429 | 2.301 ± 0.6687 | 1.835 ± 0.3406 |
| Dopamine | 8.203 ± 0.7787 | 35.27 ± 17.69 | 22.65 ± 8.602 | 23 ± 6.941 |
| Norepinephrine | 9.813 ± 1.193 | 14.9 ± 2.53 | 13.29 ± 4.336 | 12.9 ± 1.912 |

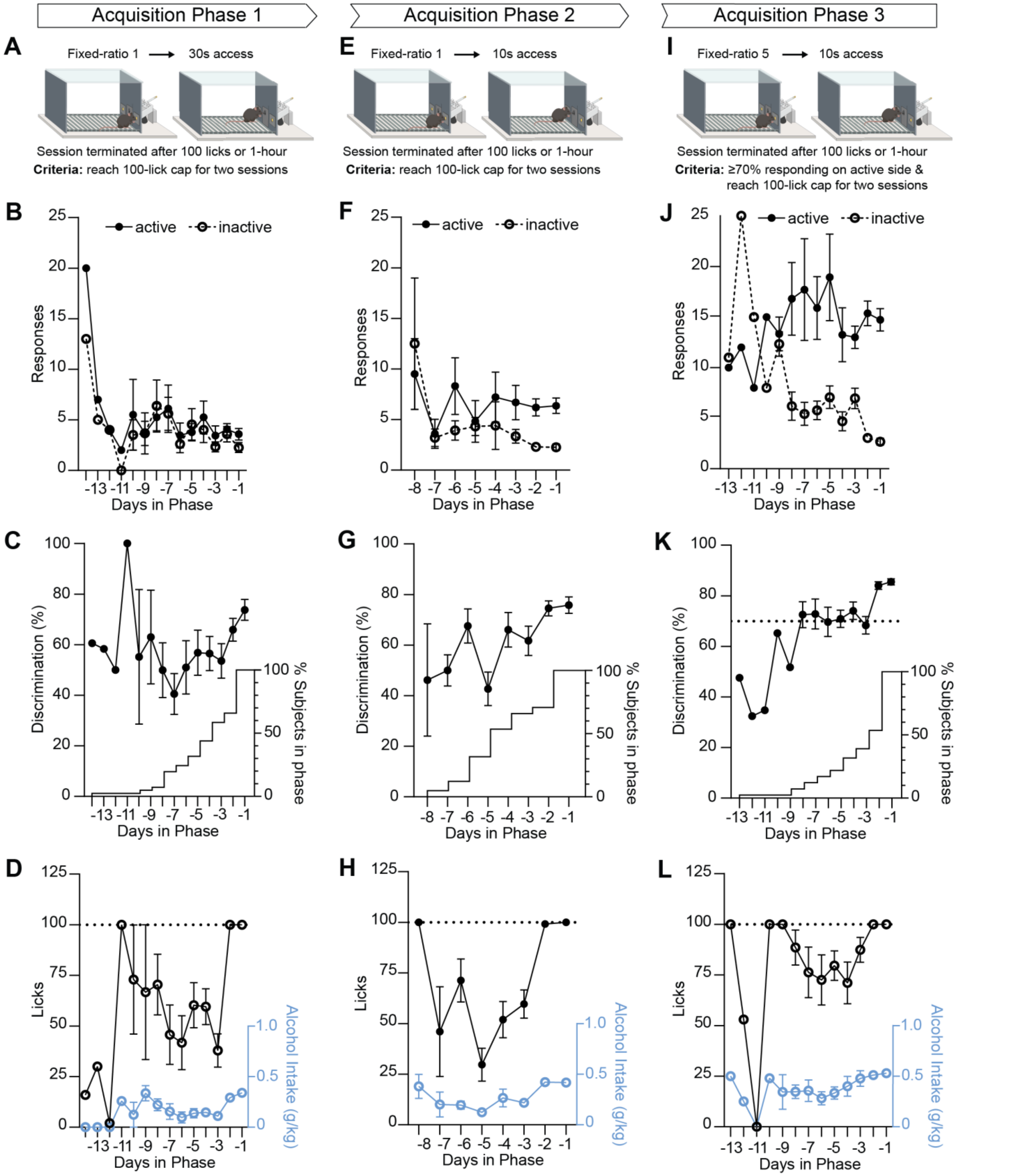


**Figure S1. Structured tracking of alcohol reinforcement acquisition training in male subjects.** Acquisition data using a phased acquisition criterion, aligned to each subjects' final session in each phase. **(A)**

Acquisition Phase 1 experimental parameters and acquisition criteria. Responses are reinforced under a fixed-ratio 1 schedule by extension of the alcohol sipper for 30s. Sessions are terminated after 100 licks are reached on the lickometer or after one hour, whichever comes first. **(B)** Active (solid line) and inactive (dotted line) responses during Acquisition Phase 1. **(C)** Percent active operandum discrimination (active responses / [active + inactive responses]) during Acquisition Phase 1. Percentage of subjects remaining for each day is indicted on the right y-axis. **(D)** Licks and alcohol Consumption (g/kg) during Acquisition Phase 1. **(E)** Acquisition Phase 2 experimental parameters and acquisition criteria. Responses are reinforced under a fixed-ratio 1 schedule by extension of the alcohol sipper for 10s. Sessions are terminated after 100 licks are reached on the lickometer or after one hour, whichever comes first. **(F)** Total nose pokes on either the active (solid line) or inactive (dotted line) nose-poke during Acquisition Phase 2. **(G)** Percent active operandum discrimination during Acquisition Phase 2. Percentage of subjects remaining for each day is indicted on the right y-axis. **(H)** Licks and alcohol Consumption (g/kg) during Acquisition Phase 2. **(I)** Acquisition Phase 3 experimental parameters and acquisition criteria. Responses are reinforced under a fixed-ratio 5 schedule by extension of the alcohol sipper for 10s. Sessions are terminated after 100 licks are reached on the lickometer or after one hour, whichever comes first. **(J)** Total nose pokes on either the active (solid line) or inactive (dotted line) nose-poke during Acquisition Phase 3. **(K)** Percent active operandum discrimination during Acquisition Phase 3 – dotted line indicates response discrimination criteria in this phase. Percentage of subjects remaining for each day is indicted on the right y-axis. **(L)** Licks and alcohol intake (g/kg) during Acquisition Phase 3. N = 41, percent of the 41 subjects represented by each data point are indicated on the right y-axis of panels C, G, and K. Error bars indicate SEM.

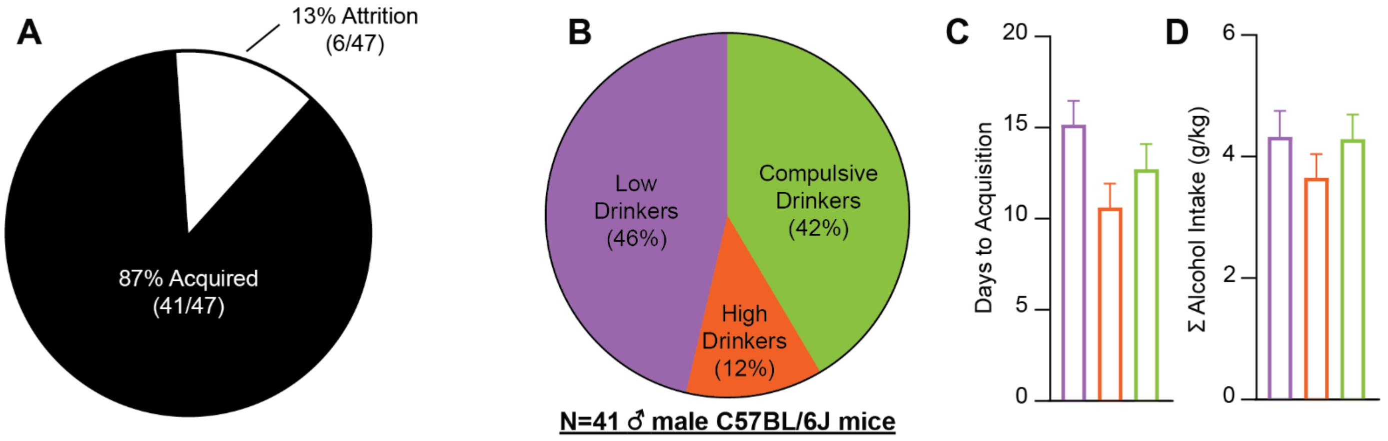


**Figure S2. Summary of acquisition data in male subjects. (A)** Of 47 animals, only six did not reach acquisition criteria. **(B)** Breakdown of phenotype membership among the 41 subjects that reached criteria and thus went on to complete self-administration experiments. **(C)** Phenotypes did not differ in average days/sessions to complete all acquisition criteria (one-way ANOVA, $F_{(2, 38)}$ = 1.743, p = 0.1887). **(D)** Cumulative alcohol consumption throughout all acquisition sessions did not differ between phenotypes (one-way ANOVA, $F_{(2, 38)}$ = 0.3287, p=0.7219). Error bars indicate SEM.

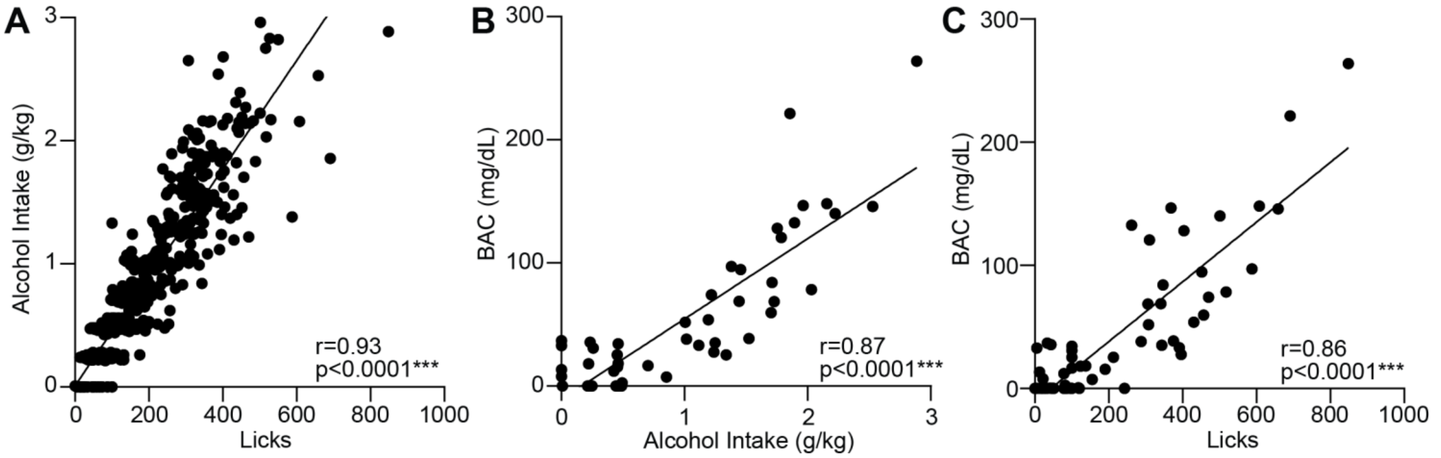


**Figure S3. Intercorrelation between licks, alcohol intake, and blood alcohol concentration. (A)** Total licks registered on the lickometer during each self-administration session were highly correlated with alcohol consumed calculated from the volumetric measures taken at the start and end of each session (n=637 sessions). **(B-C)** Blood alcohol concentration (BAC) was highly correlated with both licks and consumption (n=66 sessions).

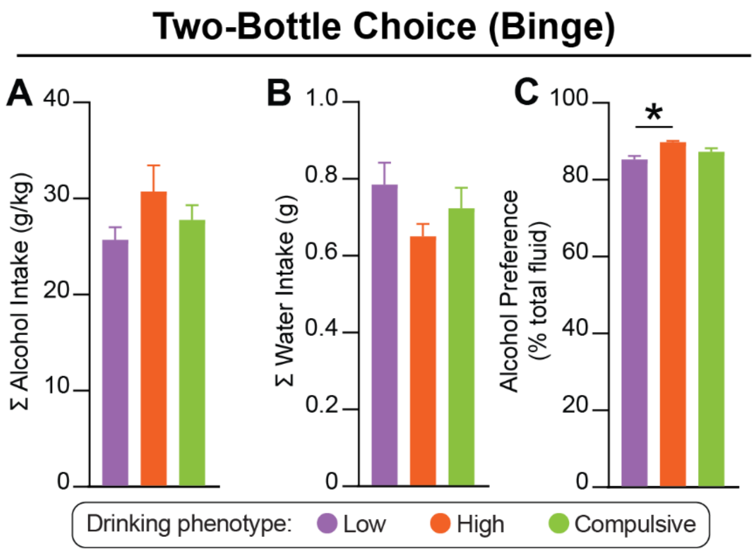


**Figure S4. Total fluid consumption and preference during two-bottle choice in male subjects. (A)** Total alcohol intake summed throughout the ten two-bottle choice sessions did not differ between phenotypes (one-way ANOVA, $F_{(2, 38)} = 1.543$, $p = 0.2269$). **(B)** Total water intake summed throughout the ten two-bottle choice sessions did not differ between phenotypes (one-way ANOVA, $F_{(2, 38)} = 0.8435$, $p=0.4381$). **(C)** High Drinkers showed greater preference for alcohol over water than Low Drinkers (one-way ANOVA, $F_{(2, 38)}= 3.458$, $p=0.0417$; Tukey's test: Low Drinkers vs High Drinkers *$p<0.05$). Low Drinkers, n=19; High Drinkers, n=5; Compulsive Drinkers, n=17. Error bars indicate SEM.

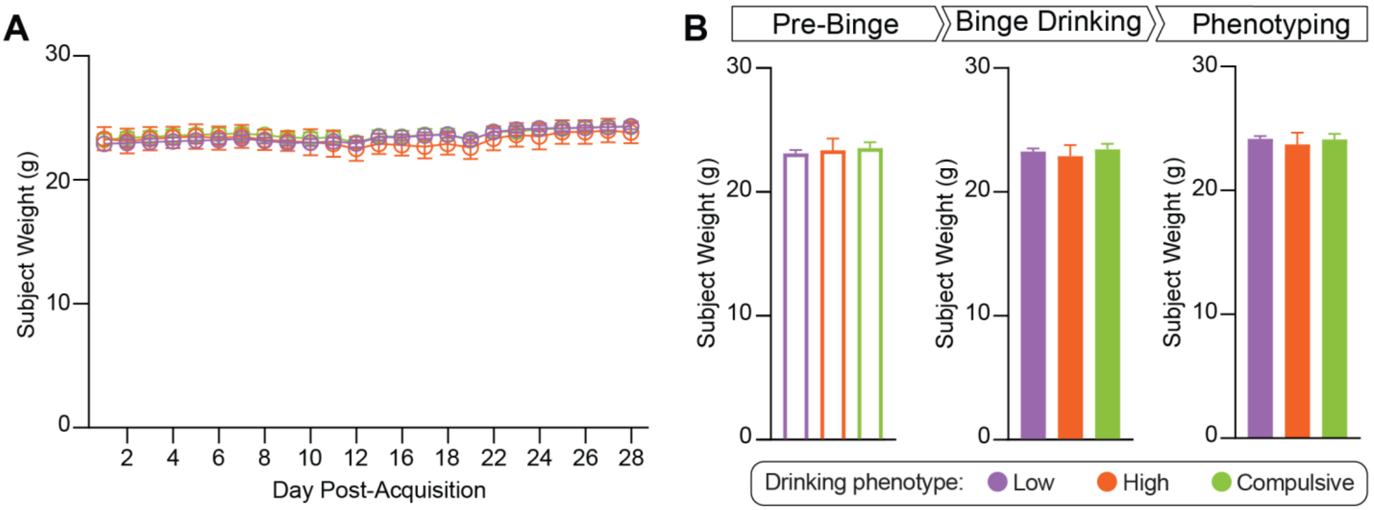


**Figure S5. Body weight does not differ between phenotypes in male mice. (A)** Body weight over the 24 daily sessions of the STAR procedure. **(B)** Average bodyweights did not differ by phenotype during the 7-day Pre-Binge period (one-way ANOVA, $F_{(2, 38)} = 0.3047$, $p = 0.7391$), the 14-day Binge period (one-way ANOVA, $F_{(2, 38)} = 0.1652$, $p = 0.8483$), or the 7-day Phenotyping period (one-way ANOVA, $F_{(2, 38)} = 0.2498$, $p = 0.7802$). Low Drinkers, n=19; High Drinkers, n=5; Compulsive Drinkers, n=17. Error bars indicate SEM.

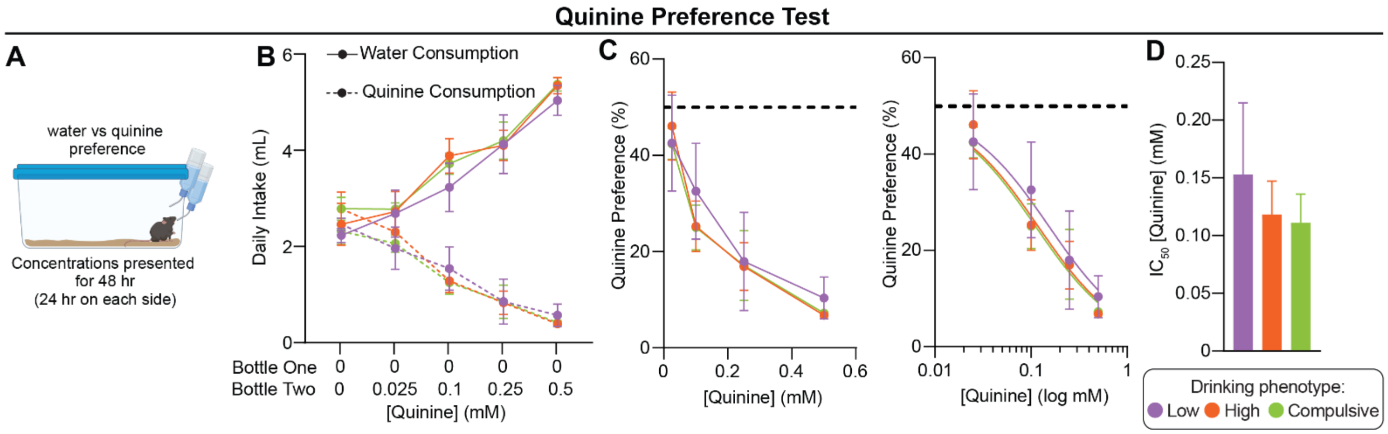


**Figure S6. Phenotypes do not differ in sensitivity to quinine in a taste preference assay in male mice.** **(A)** Schematic of experimental setup. Animals were presented with two bottles containing water or quinine adulterated water. Bottles were weighed every 24 hours to determine fluid intake and switched to the opposite side to account for side preferences. Each concentration of quinine was presented once in each bottle position, for a total of 48 hours, and values were averaged to determine taste preference at each concentration. **(B)** Daily average intake of water and quinine adulterated water across a quinine concentration response curve. **(C)** Quinine preference, calculated as the percent of total fluid intake drank from the quinine bottle, across a quinine concentration response curve plotted on a linear (left) or logarithmic (right) scale. **(D)** The $IC_{50}$ of quinine to produce taste avoidance (i.e. the concentration of quinine required to produced half-maximal avoidance) did not differ between phenotypes (one-way ANOVA, $F_{(2, 12)} = 0.3375$, $p = 0.7201$). Low Drinkers, n=4; High Drinkers, n=5; Compulsive Drinkers, n=6. Error bars indicate SEM.

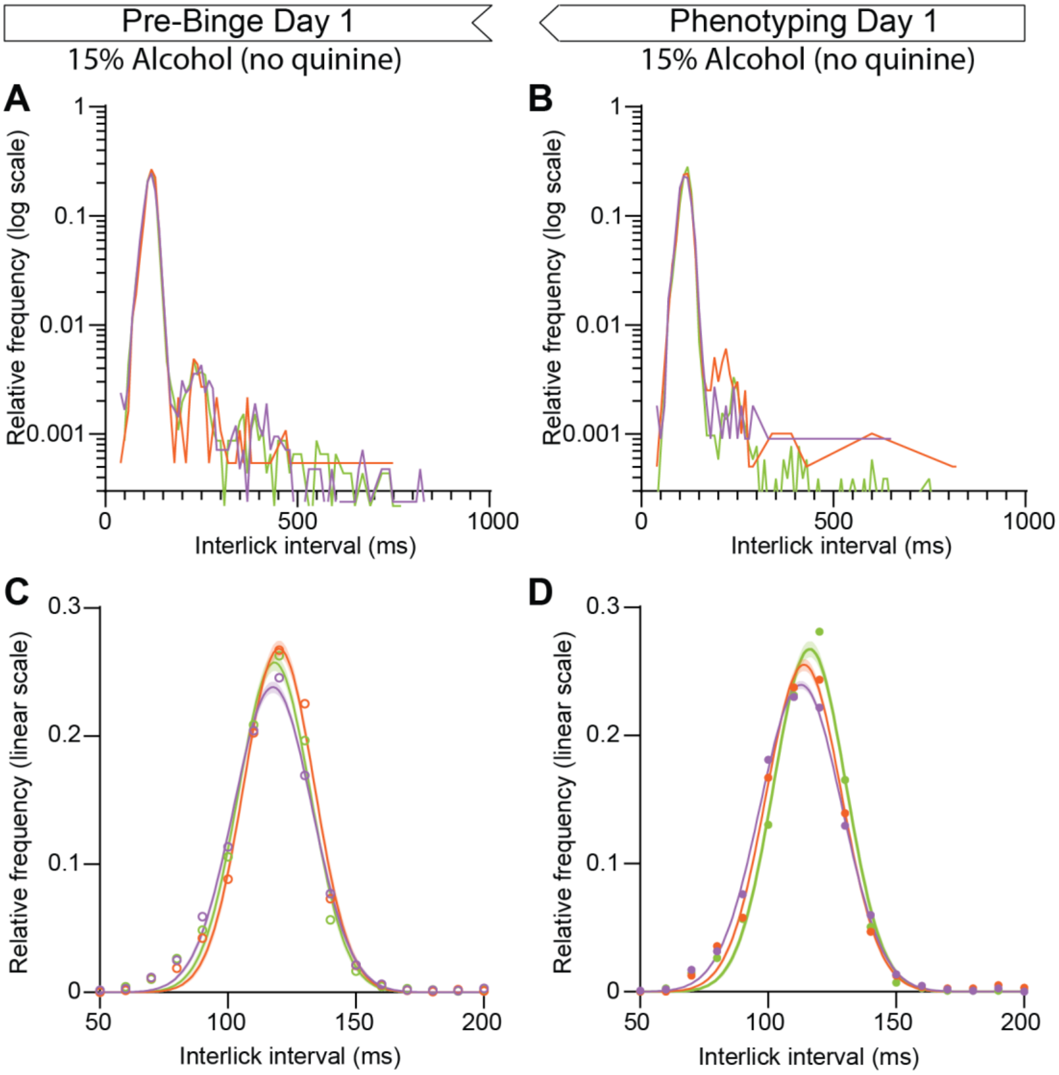


**Figure S7. Lick microstructural patterns during alcohol self-administration in male mice.** Interlick interval was determined for all licks during the first alcohol self-administration session of the Pre-Binge and STAR phenotyping periods. **(A-B)** Interlick interval distributions by phenotype plotted as a fraction of total licks and plotted on a log **(A)** or linear **(B)** scale revealed that High Drinkers displayed a different distribution compared to both Low Drinkers and Compulsive Drinkers (Kruskal-Wallis test, $H_{(3)} = 8.146$, $p = 0.0170$; Dunn's multiple comparisons test: Low Drinkers vs High Drinkers *$p<0.05$). **(C-D)** During the first session of the Phenotyping sessions (STAR Phenotyping day 1) alcohol self-administration, interlick interval distributions by phenotype plotted as a fraction of total licks and plotted on a log **(C)** or linear **(D)** scale revealed that High Drinkers no longer differed from Low Drinkers. However, High Drinkers displayed a different distribution compared to Compulsive Drinkers (Kruskal-Wallis test, $H_{(3)} = 8.310$, $p = 0.0157$; Dunn's multiple comparisons test: High Drinkers vs Compulsive Drinkers *$p<0.05$).

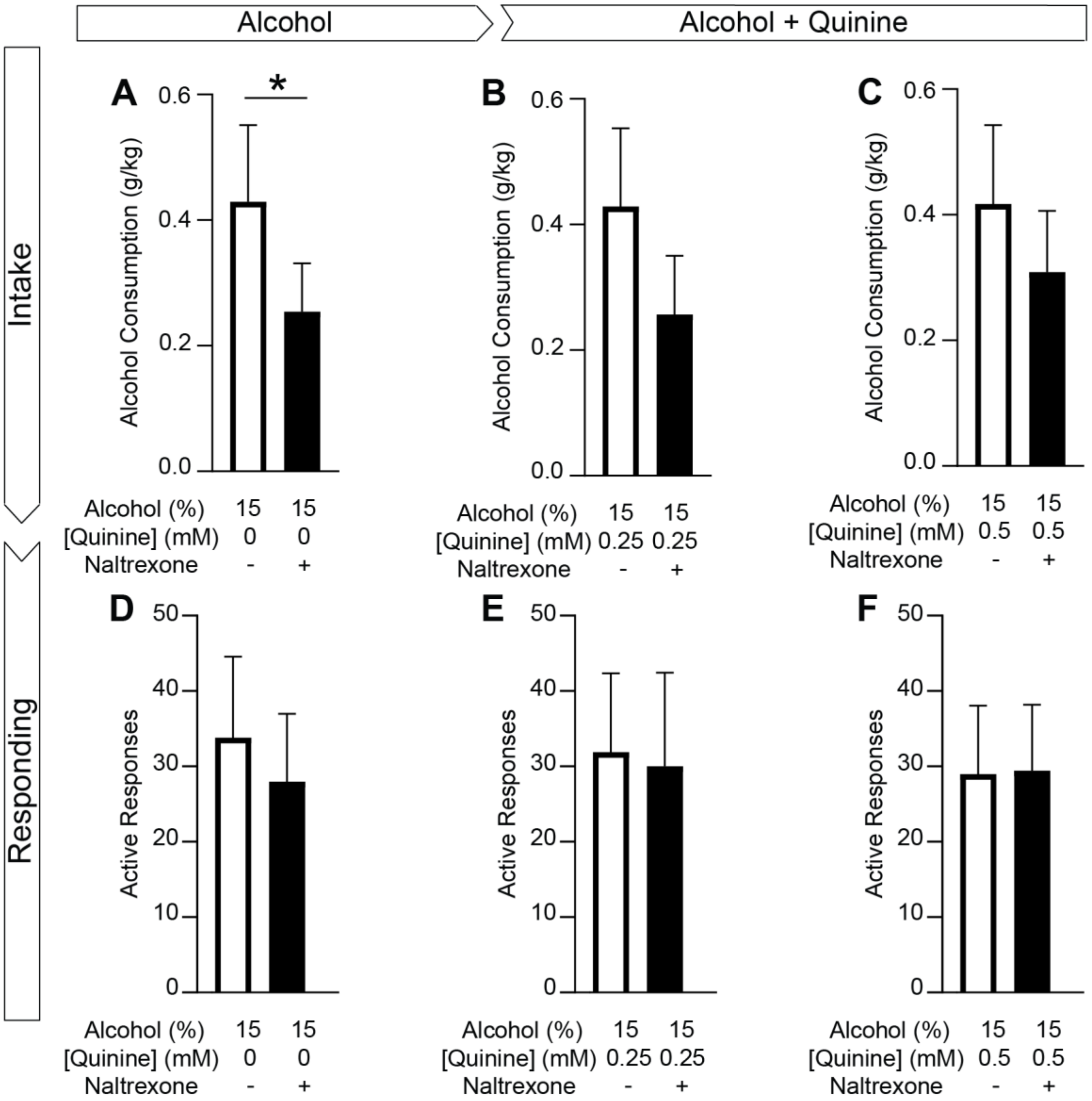


**Figure S8. Treatment with naltrexone decreases unpunished alcohol consumption, but not responding in male subjects. (A)** Alcohol consumption is reduced following administration of 1 mg/kg naltrexone as compared to saline control (paired t-test, $t_{23} = 2.259$, $p = 0.0337$). **(B)** Consumption of alcohol adulterated with 250μM quinine is not reduced following naltrexone administration compared to saline (paired t-test, $t_{23} = 1.634$, $p = 0.1158$). **(C)** Consumption of alcohol adulterated with 500μM quinine is not reduced following naltrexone administration compared to saline (paired t-test, $t_{23} = 1.134$, $p = 0.2685$). **(D)** Responding for alcohol is not reduced following naltrexone administration compared to saline (paired t-test, $t_{23} = 1.137$, $p = 0.2674$). **(E)** Responding for alcohol adulterated with 250μM quinine is not reduced following naltrexone administration compared to saline (paired t-test, $t_{23} = 0.1676$, $p = 0.8684$). **(F)** Responding for alcohol adulterated with 500μM is not reduced following naltrexone administration compared to saline (paired t-test, $t_{23} = 0.07617$, $p = 0.9399$). $*p < 0.05$. N = 24. Error bars indicate SEM.

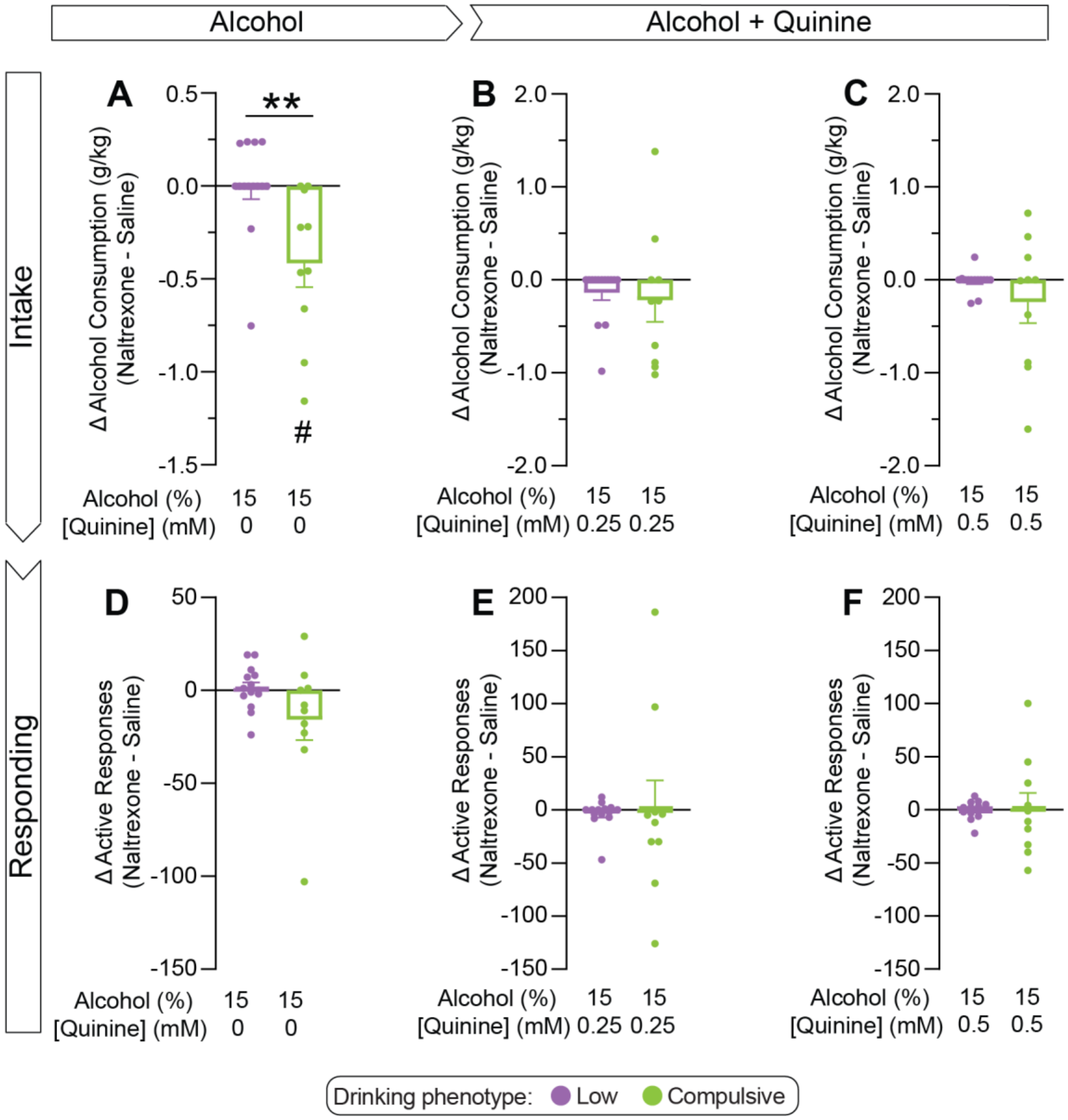


**Figure S9. Differential effects of naltrexone on alcohol consumption in low and compulsive drinking phenotypes.** Change in each measure induced by 1 mg/kg naltrexone was calculated for each session type (naltrexone session minus respective saline session). Note that this cohort did not contain any High Drinkers. **(A)** For the alcohol only session, Compulsive Drinkers displayed reduced intake relative to zero (one sample t-test, $H_0 = 0$, $t_9 = 3.242$, p = 0.0101) while Low Drinkers did not (one sample t-test, $H_0 = 0$, $t_{13} = 0.04524$, p = 0.9646), as well as a reduced intake compared to directly to Low Drinkers (unpaired t-test, $t_{22} = 3.069$, p = 0.0056). **(B)** Naltrexone treatment did not induce a change in consumption of alcohol adulterated with 250µM quinine in either Low or Compulsive Drinkers compared to zero (Low Drinkers, one sample t-test, $H_0 = 0$, $t_{13} = 1.746$, p = 0.1045; Compulsive Drinkers, one sample t-test, $H_0 = 0$, $t_9 = 0.9309$, p = 0.3762) and did not have a differential effect between phenotypes (unpaired t-test, $t_{22} = 0.3590$, p = 0.7230). **(C)** Naltrexone treatment did not induce a change in consumption of alcohol adulterated with 500µM quinine in either Low or Compulsive Drinkers compared to zero (Low Drinkers, one sample t-test, $H_0 = 0$, $t_{13} = 0.5098$, p = 6187; Compulsive Drinkers, one sample t-test, $H_0 = 0$, $t_9 = 1.056$, p = 0.3185) and did not have a differential effect between phenotypes (unpaired t-test, $t_{22} = 1.158$, p = 0.2595). **(D)** Naltrexone treatment did not induce a change in responding for alcohol in either Low or Compulsive Drinkers compared to zero (Low Drinkers, one sample t-

test, $H_0 = 0$, $t_{13} = 0.3904$, $p = 0.7025$; Compulsive Drinkers, one sample t-test, $H_0 = 0$, $t_9 = 1.413$, $p = 0.1913$) and did not have a differential effect between phenotypes (unpaired t-test, $t_{22} = 1.689$, $p = 0.1054$). **(E)** Naltrexone treatment did not induce a change in responding for alcohol adulterated with 250μM quinine in either Low or Compulsive Drinkers compared to zero (Low Drinkers, one sample t-test, $H_0 = 0$, $t_{13} = 0.9882$, $p = 0.3411$; Compulsive Drinkers, one sample t-test, $H_0 = 0$, $t_9 = 0.01838$, $p = 0.9857$) and did not have a differential effect between phenotypes (unpaired t-test, $t_{22} = 0.1756$, $p = 0.8622$). **(F)** Naltrexone treatment did not induce a change in responding for alcohol adulterated with 500μM quinine in either Low or Compulsive Drinkers compared to zero (Low Drinkers, one sample t-test, $H_0 = 0$, $t_{13} = 0.09437$, $p = 0.9263$; Compulsive Drinkers, one sample t-test, $H_0 = 0$, $t_9 = 0.09628$, $p = 0.9254$) and did not have a differential effect between phenotypes (unpaired t-test, $t_{22} = 0.1294$, $p = 0.8982$). ** $p < 0.01$ between groups; # $p < 0.05$ vs 0. Low Drinkers n = 10; Compulsive Drinkers n = 14. Error bars indicate SEM.

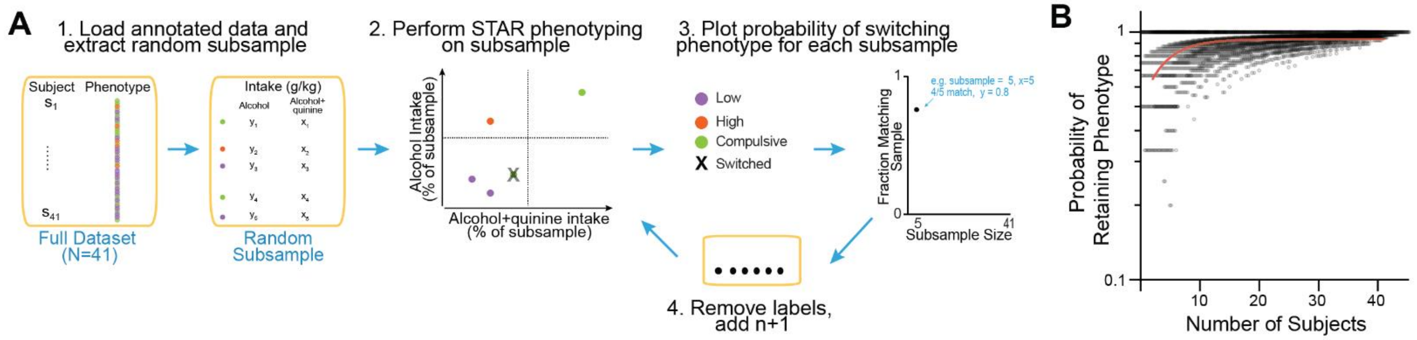


**Figure S10. Permutation test of phenotype stability and recommended minimum sample size for STAR phenotyping.** Any individual differences-based quantification is made in relation to a population or sample, and therefore phenotypes can shift between different samples. Here we sought to empirically determine the sensitivity of STAR phenotyping to sample size by randomly resampling and phenotyping with varying samples sizes. **(A)** Pipeline: **(1)** Data from all male subjects was loaded. Each subject was labeled with their assigned phenotype from the full dataset, as well as the two g/kg values that were entered to derive these assignments (average intake during the three alcohol only sessions and average intake on the four alcohol+quinine sessions). A random subset of subjects was extracted from the dataset (a subsample of n=5 is depicted in the example). Phenotype labels were removed and each subject was associated only with their average intake over the three alcohol only sessions ($Y_n$) and over the four alcohol+quinine sessions ($X_n$). **(2)** Next, STAR phenotyping was performed using the same equation as usual but only subjects in the randomly selected subset were included [alcohol value = (subject mean session 1-3 (g/kg)/mean of all subjects day 1-3 (g/kg))*100; alcohol+quinine value = (subject mean session 4-7 (g/kg)/mean of all subjects day 4-7 (g/kg))*100]. Subjects within the subsample were then assigned a phenotype based on these values. **(3)** Each subject in the subsample was then compared to determine if the assigned phenotype matched or differed from the phenotype label in the full dataset, and the probability of retaining the same phenotype was determined [number of matched subjects / subjects in the subsample] and plotted. **(4)** Phenotype assignments were again removed, and one additional subject was added to the subsample. Steps 2-4 were repeated such that probability of phenotype switching was determined using subsamples of 2 through 41 subjects. The resampling pipeline was iterated 100 times to create a distribution of the probability of obtaining the same phenotype assignment across a range of sample sizes (sample sizes 2 through 41, 100 iterations each for a total of 4,000 values in the final distribution; see **Supplemental Video 1** for visualization of pipeline). **(B)** Probabilities calculated at each sample size for each iteration are plotted in grey (note that overlapping values are staggered horizontally to allow visualization) and the distribution was fit with a Gompertz growth curve, plotted in red, to estimate the asymptotes and rate constant K. Best-fit values revealed a rate constant of K=0.2606 (95% confidence interval: 0.2452 to 0.2774) and an inflection point (1/K) at x=3.84 (95% confidence interval: 3.605 to 4.078), which approximates 36% of growth to reach the upper asymptote (Winsor 1932; Tjørve and Tjørve 2017). The ordinate at 3/K (x=11.51) was >95% of the asymptote; thus, a n=12 was the minimum sample size needed to retain phenotype stability with an $\alpha$ of 0.05 or less. Exact power requirements will vary by laboratory and experimental question, we recommend starting with a conservative minimum sample size of at least N=15 (≈4/K in our dataset), tested under identical experimental conditions, before STAR phenotyping is performed.

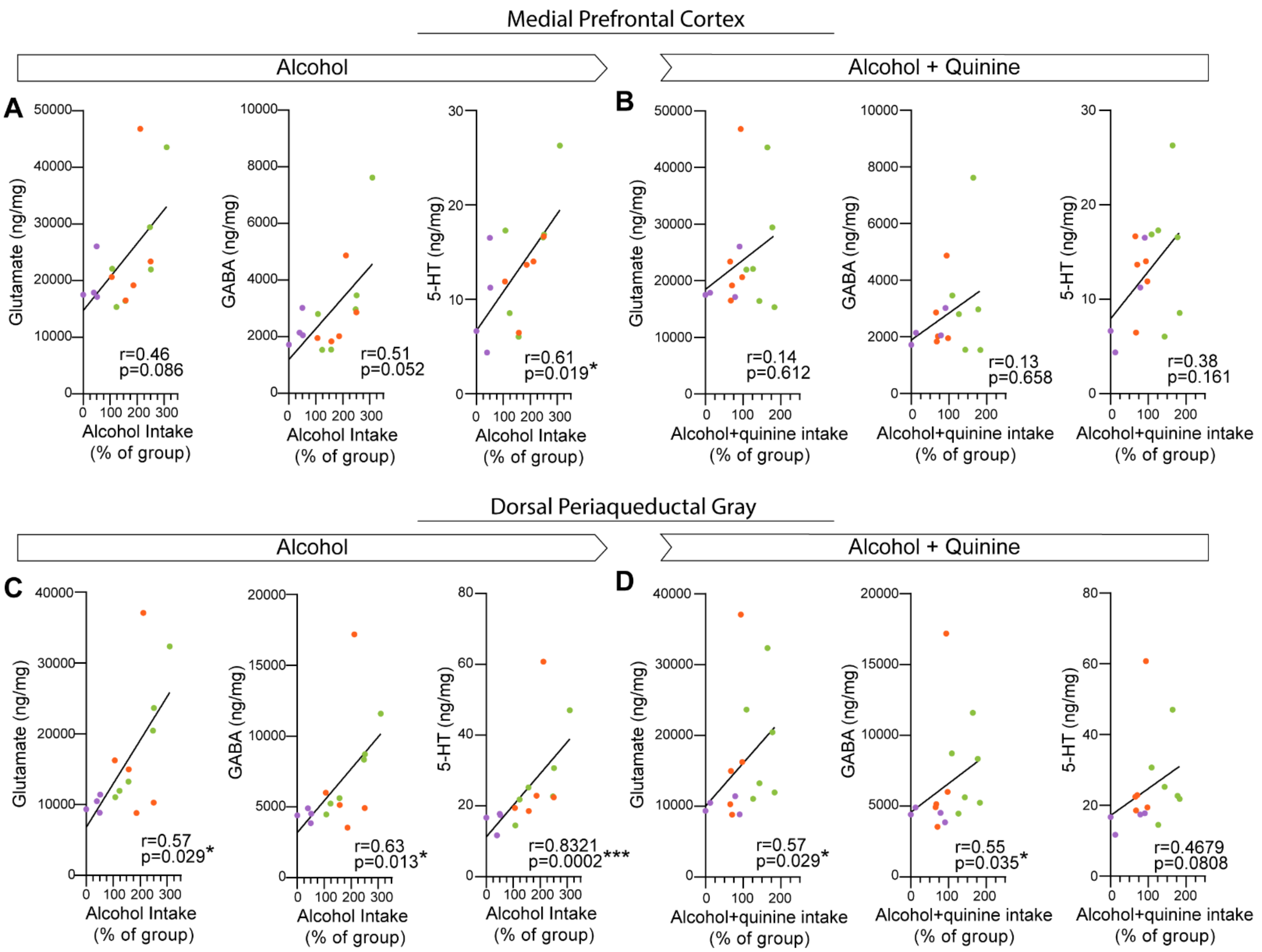


**Figure S11. Association between mPFC and dPAG biogenic amine concentrations and STAR phenotyping measures.** A subset of the data in Figure 5 is replotted here to provide visualization of individual animals and phenotypes. **(A)** Correlations between mPFC concentrations of glutamate, GABA, and 5-HT with unpunished alcohol intake values. 5-HT is positively correlated with alcohol intake when unpunished (i.e. high drinking trait). **(B)** Correlations between mPFC concentrations of glutamate, GABA, and 5-HT with alcohol+quinine intake. None of the analytes in mPFC are correlated with alcohol+quinine intake (i.e. compulsive drinking trait). **(C)** Correlations between dPAG concentrations of glutamate, GABA, and 5-HT with unpunished alcohol intake values. All three analytes are positively correlated with alcohol intake when unpunished (i.e. high drinking trait). **(D)** Correlations between dPAG concentrations of glutamate, GABA, and 5-HT with alcohol+quinine intake. dPAG concentrations of glutamate and GABA, but not 5-HT, are positively correlated with alcohol+quinine intake (i.e. compulsive drinking trait). Spearman's correlation coefficient: *p < 0.05; **p < 0.01; ***p < 0.001. Low Drinkers, n=4; High Drinkers, n=5; Compulsive Drinkers, n=6.

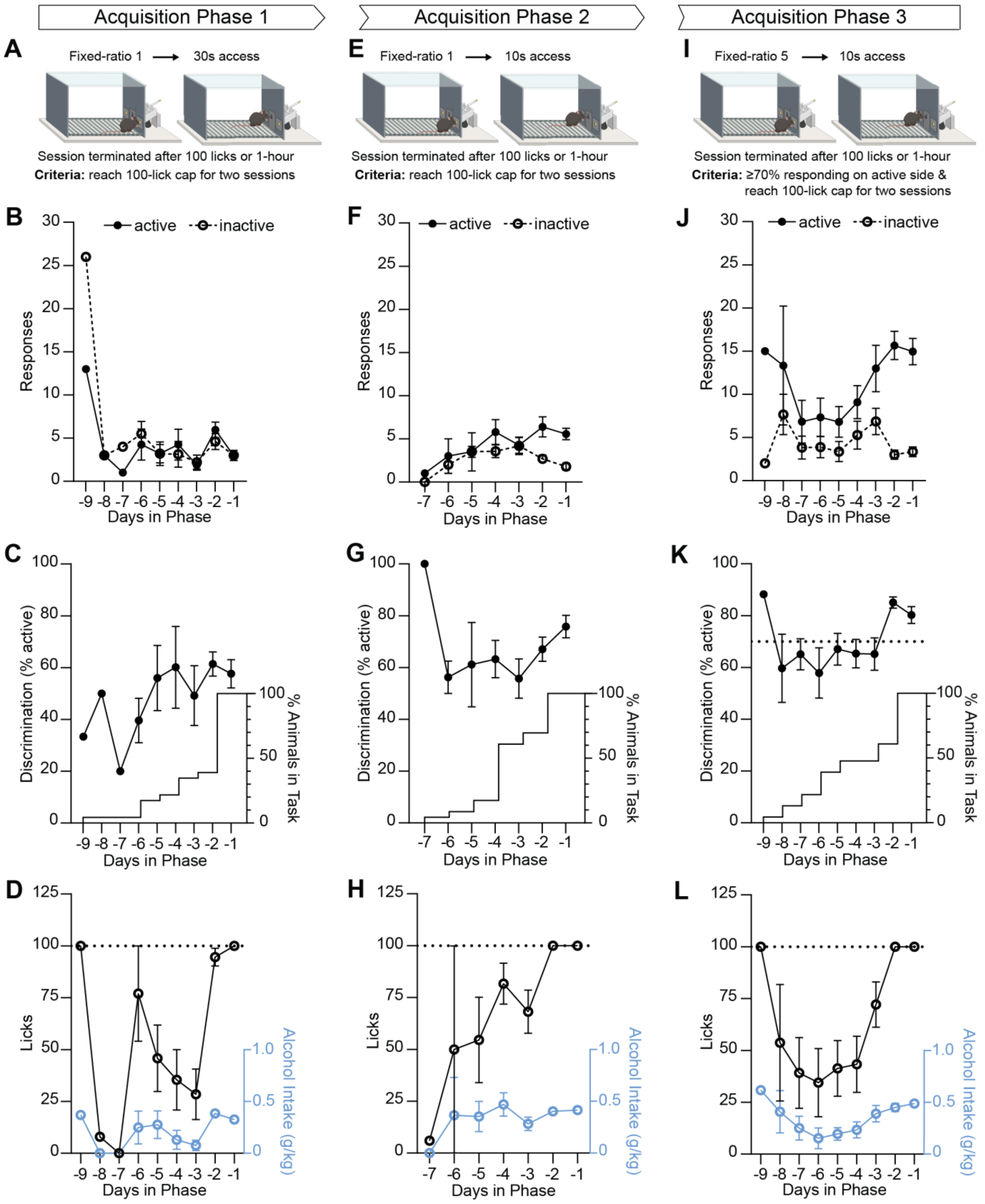


**Figure S12. Structured tracking of alcohol reinforcement acquisition training in female subjects.** Acquisition data using a phased acquisition criterion, aligned to each subjects' final session in each phase. **(A)**

Acquisition Phase 1 experimental parameters and acquisition criteria. Responses are reinforced under a fixed-ratio 1 schedule by extension of the alcohol sipper for 30s. Sessions are terminated after 100 licks are reached on the lickometer or after one hour, whichever comes first. **(B)** Active (solid line) and inactive (dotted line) responses during Acquisition Phase 1. **(C)** Percent active operandum discrimination (active responses / [active + inactive responses]) during Acquisition Phase 1. Percentage of subjects remaining for each day is indicted on the right y-axis. **(D)** Licks and alcohol consumption (g/kg) during Acquisition Phase 1. **(E)** Acquisition Phase 2 experimental parameters and acquisition criteria. Responses are reinforced under a fixed-ratio 1 schedule by extension of the alcohol sipper for 10s. Sessions are terminated after 100 licks are reached on the lickometer or after one hour, whichever comes first. **(F)** Total nose pokes on either the active (solid line) or inactive (dotted line) nose-poke during Acquisition Phase 2. **(G)** Percent active operandum discrimination during Acquisition Phase 2. Percentage of subjects remaining for each day is indicted on the right y-axis. **(H)** Licks and alcohol consumption (g/kg) during Acquisition Phase 2. **(I)** Acquisition Phase 3 experimental parameters and acquisition criteria. Responses are reinforced under a fixed-ratio 5 schedule by extension of the alcohol sipper for 10s. Sessions are terminated after 100 licks are reached on the lickometer or after one hour, whichever comes first. **(J)** Total nose pokes on either the active (solid line) or inactive (dotted line) nose-poke during Acquisition Phase 3. **(K)** Percent active operandum discrimination during Acquisition Phase 3 – dotted line indicates response discrimination criteria in this phase. Percentage of subjects remaining for each day is indicted on the right y-axis. **(L)** Licks and alcohol consumption (g/kg) during Acquisition Phase 3. N = 23, percent of the 23 subjects represented by each data point are indicated on the right y-axis of panels C, G, and K. Error bars indicate SEM.

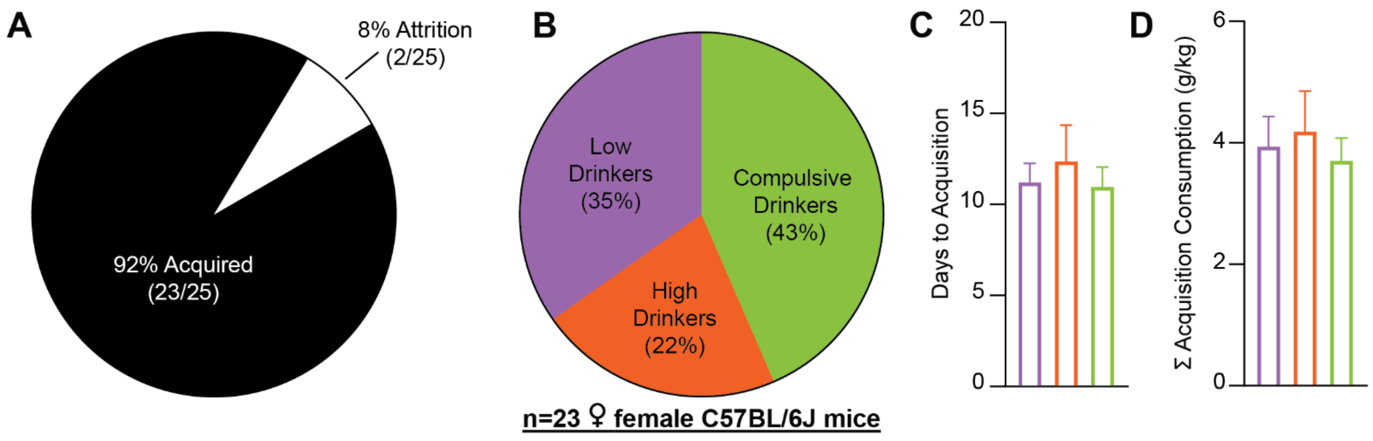


**Figure S13. Summary of acquisition data in female subjects. (A)** Of 25 animals, only two did not reach acquisition criteria. **(B)** Breakdown of phenotype membership among the 23 subjects that reached criteria and thus went on to complete self-administration experiments. **(C)** Phenotypes did not differ in average days/sessions to complete all acquisition criteria (one-way ANOVA, $F_{(2, 20)}$ = 0.2888, p = 0.7522). **(D)** Cumulative alcohol consumption throughout all acquisition sessions does not differ between phenotypes (one-way ANOVA, $F_{(2, 20)}$ = 0.2441, p = 0.7857). Error bars indicate SEM.

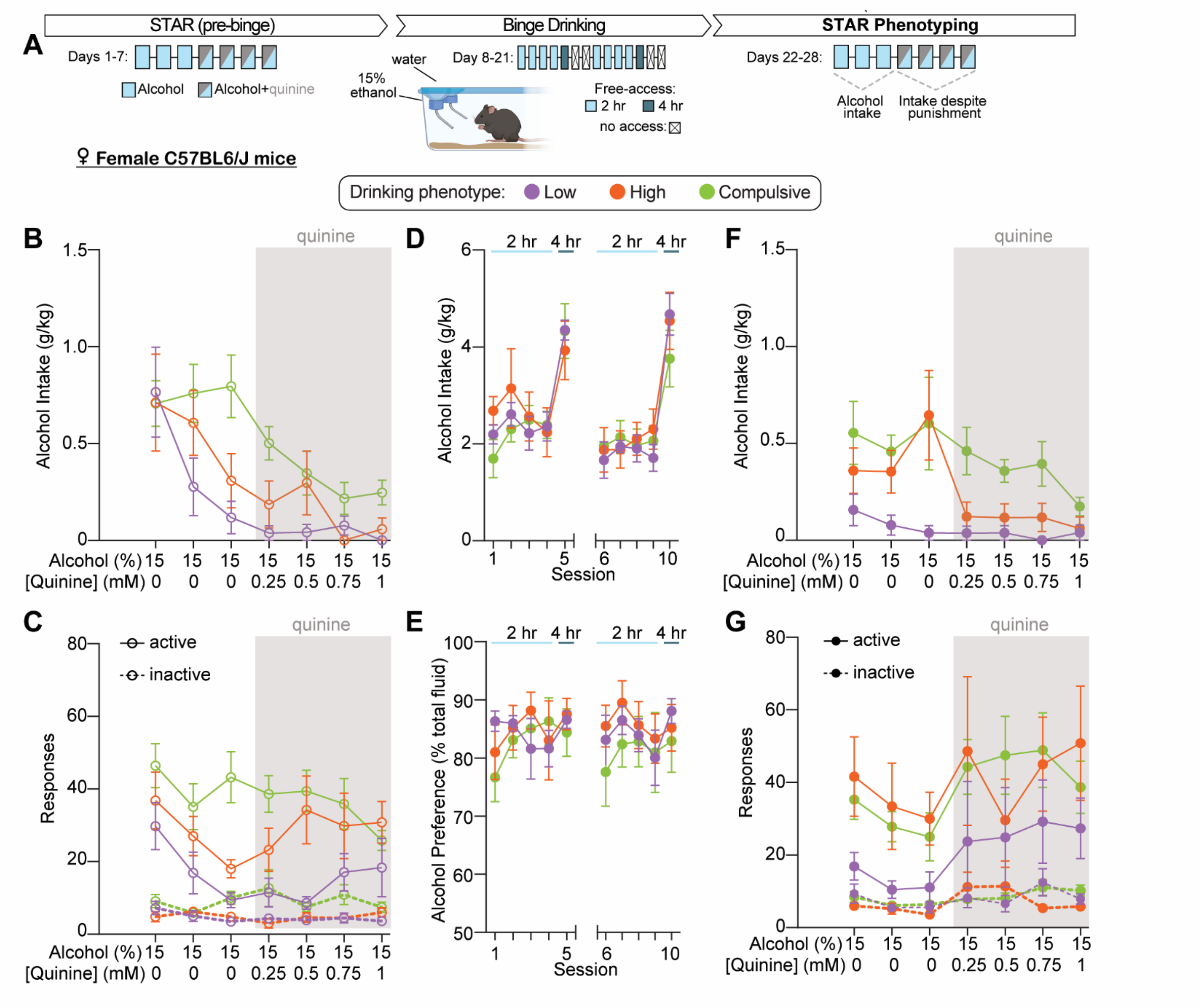


**Figure S14. STAR procedure allows assessment of experience-dependent phenotype dynamics in female subjects. (A)** Experimental timeline. Female subjects were tested under the same conditions as the initial studies in male subjects presented in Figure 1. Group assignments are assigned based on STAR phenotyping from session following the binge drinking period. **(B-C)** Alcohol intake **(B)** and operant responding **(C)** across three alcohol self-administration sessions. Graded concentrations of quinine are then added to the alcohol solution across sessions to test the sensitivity of alcohol reinforcement to punishment. **(D-E)** Subsequently, animals are allowed to free access to alcohol during a two-bottle choice procedure, which engenders high levels of alcohol consumption **(D)** and alcohol preference compared to water **(E)**. **(F-G)** STAR Phenotyping self-administration sessions demonstrated wide individual differences in alcohol intake with and without quinine punishment **(F)** as well as operant responding for alcohol access **(G)**. Low Drinkers, n=8; High Drinkers, n=5; Compulsive Drinkers, n=10. Error bars indicate SEM.

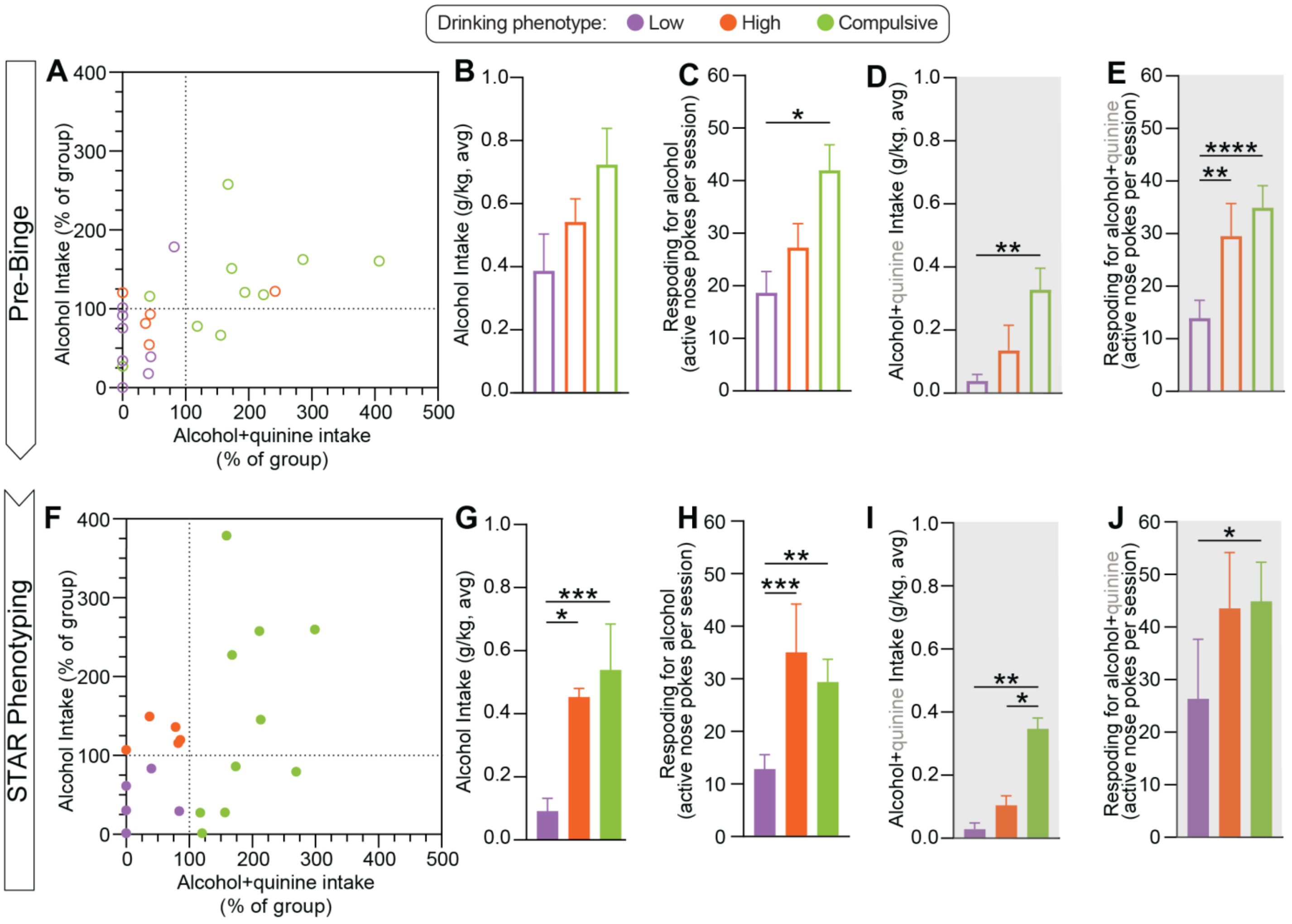


**Figure S15. Phenotypic alcohol drinking behaviors emerge over time and experience in female subjects. (A) Pre-Binge:** normalized distributions of alcohol intake (y-axis) and alcohol intake during quinine sessions (x-axis) from pre-binge self-administration sessions. **(B)** Intake over the three pre-binge alcohol only self-administration sessions does not differ by phenotype (nested one-way ANOVA, $F_{(2,6)} = 2.106$, $p = 0.2028$; 3 groups, 3 days per group, 67 total values). **(C)** Active nose-poke responses over the three Pre-Binge alcohol only self-administration sessions differs by phenotype with Compulsive Drinkers responding more than low drinkers (nested one-way ANOVA, $F_{(2,6)} = 6.226$, $p = 0.0344$; 3 groups, 3 days per group, 67 total values). **(D)** Intake during Pre-Binge alcohol+quinine self-administration sessions differs by phenotype with greater intake in Compulsive Drinkers compared to Low Drinkers (nested one-way ANOVA, $F_{(2,9)} = 8.355$, $p = 0.0089$; 3 groups, 4 days per group, 92 total values). **(E)** Active nose-poke responses during Pre-Binge alcohol+quinine self-administration sessions differs by phenotype, with High and Compulsive Drinkers displaying high response rates compared to Low Drinkers (nested one-way ANOVA, $F_{(2,89)} = 15.48$, $p < 0.0001$; 3 groups, 4 days per group, 92 total values). **(F) STAR Phenotyping:** normalized distributions of alcohol intake (y-axis) and alcohol intake during quinine sessions (x-axis) from STAR Phenotyping self-administration sessions. **(G)** High and Compulsive Drinkers display greater alcohol intake over the three STAR Phenotyping alcohol only self-administration sessions compared to Low Drinkers (nested one-way ANOVA, $F_{(2, 66)} = 8.593$, $p = 0.0005$; 3 groups, 3 days per group, 69 total values). **(H)** High and Compulsive Drinkers display higher active nose-poke responses over the three STAR Phenotyping alcohol only self-administration sessions compared to Low

Drinkers (nested one-way ANOVA, $F_{(2, 66)} = 10.41$, p = 0.0001; 3 groups, 3 days per group, 69 total values). **(I)** Compulsive Drinkers have higher intake over the four STAR Phenotyping alcohol+quinine self-administration sessions compared to High and Low Drinkers (nested one-way ANOVA, $F_{(2, 9)} = 15.82$, p = 0.0011; 3 groups, 4 days per group, 92 total values). **(J)** Compulsive Drinkers display higher active nose-poke responses over the four STAR Phenotyping alcohol+quinine self-administration sessions compared to Low Drinkers (nested one-way ANOVA, $F_{(2, 89)} = 3.454$, p = 0.0359; 3 groups, 4 days per group, 92 total values). All post hoc comparisons used Tukey's test: *p < 0.05; **p < 0.01; ***p < 0.001; ****p < 0.0001. Alcohol only days: 3 groups x 3 days (Low n=8, High n=5, Compulsive n=10); alcohol+quinine days 3 groups x 4 days (Low n=8, High n=5, Compulsive n=10). Error bars indicate SEM.

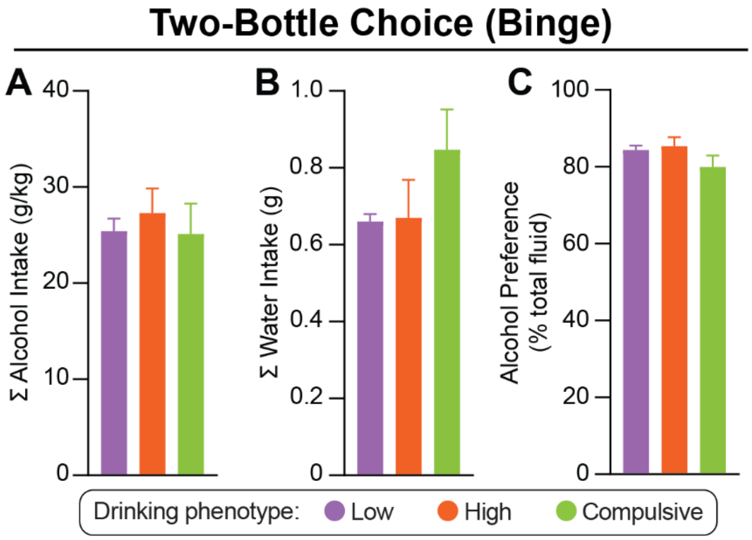


**Figure S16. Total fluid consumption and preference by phenotype during two-bottle choice in female subjects. (A)** Total alcohol intake summed throughout the ten two-bottle choice sessions does not differ between phenotypes (one-way ANOVA, $F_{(2, 20)} = 0.1457$, p=0.8653). **(B)** Total water intake summed throughout the ten two-bottle choice sessions does not differ between phenotypes (one-way ANOVA, $F_{(2, 20)} = 1.571$, p=0.2325). **(C)** Average alcohol preference throughout the ten two-bottle choice sessions does not differ between phenotypes (one-way ANOVA, $F_{(2, 20)} = 1.361$, p=0.2791). Low Drinkers, n=8; High Drinkers, n=5; Compulsive Drinkers, n=10. Error bars indicate SEM.

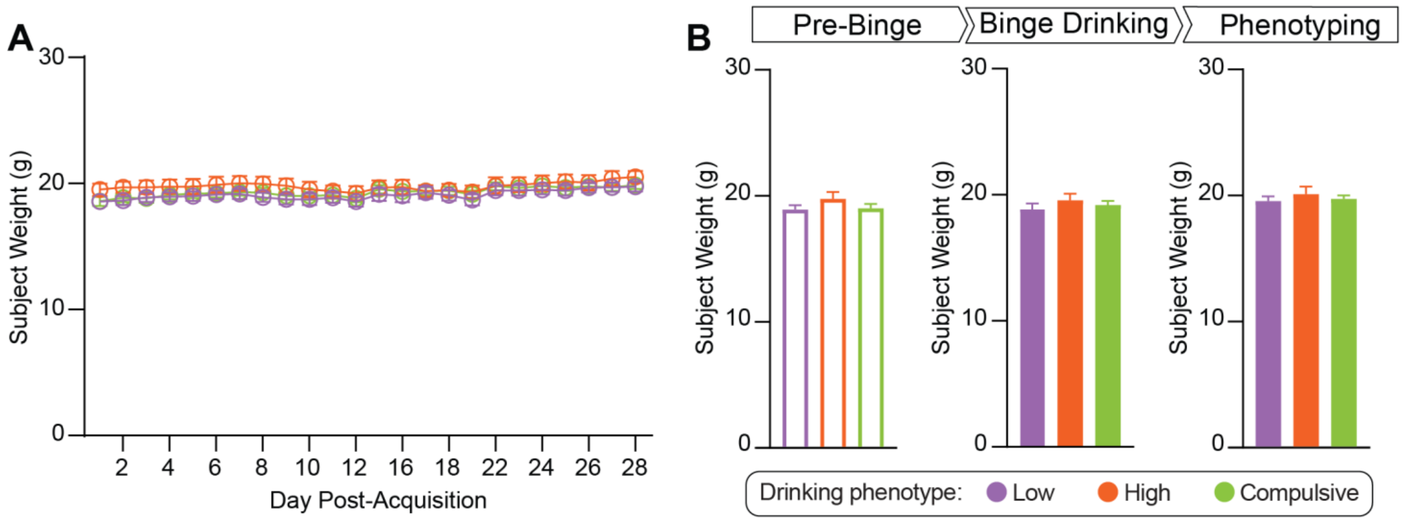


**Figure S17. Body weight does not differ between phenotypes in female subjects. (A)** Body weight over the 24 sessions pre-binge, binge, and STAR phenotyping sessions. **(B)** Average bodyweights do not differ by phenotype during the 7-day testing period prior to binge (one-way ANOVA, $F_{(2, 20)}$ = 1.079, p = 0.3588), the 14-day Binge period (one-way ANOVA, $F_{(2, 20)}$ = 0.5887, p =0.5644), or the 7-day STAR phenotyping period (one-way ANOVA, $F_{(2, 20)}$ = 0.4511, p = 0.6433). Low Drinkers, n=8; High Drinkers, n=5; Compulsive Drinkers, n=10. Error bars indicate SEM.

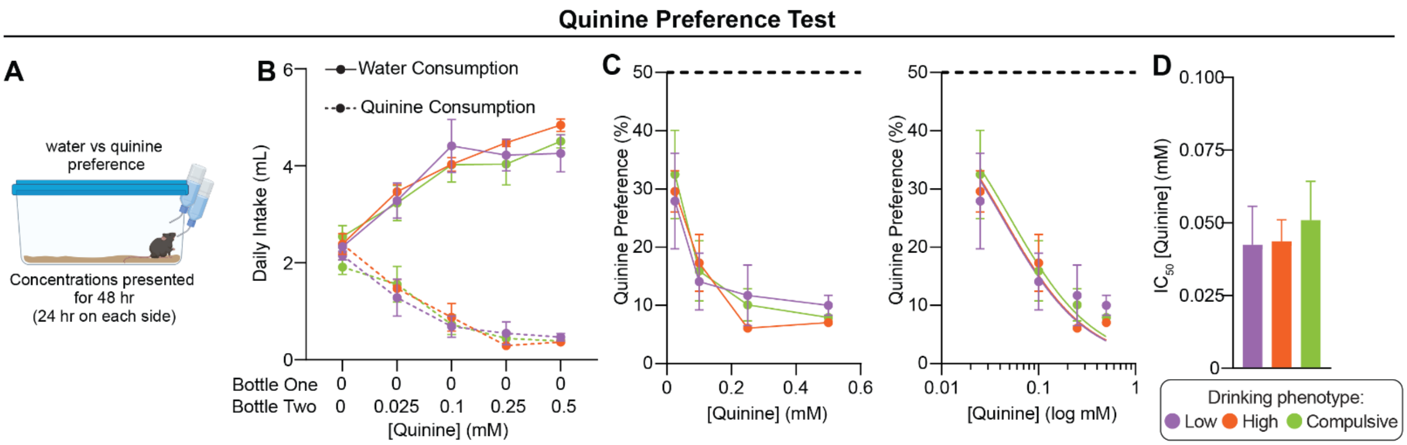


**Figure S18. Phenotypes do not differ in sensitivity to quinine in a taste preference assay in female subjects. (A)** Schematic of experimental setup. Animals are presented with two bottles containing water or quinine adulterated water. Bottles are weighed every 24 hours to determine fluid intake and switched to the opposite side to account for side preferences. Each concentration of quinine is presented once in each bottle position, for a total of 48 hours, and values are averaged to determine taste preference at each concentration. **(B)** Daily average intake of water and quinine adulterated water across a quinine concentration response curve. **(C)** Quinine preference, calculated as the percent of total fluid intake drank from the quinine bottle, across a quinine concentration response curve plotted on a linear (left) or logarithmic (right) scale. **(D)** The $IC_{50}$ of quinine to produce taste avoidance (i.e. the concentration of quinine required to produced half-maximal avoidance) did not differ between phenotypes (one-way ANOVA, $F_{(2, 12)} = 0.1577$, $p = 0.8558$). Low Drinkers, n=5; High Drinkers, n=5; Compulsive Drinkers, n=5. Error bars indicate SEM.

## References

Tjørve, Kathleen M. C., and Even Tjørve. 2017. “The Use of Gompertz Models in Growth Analyses, and New Gompertz-Model Approach: An Addition to the Unified-Richards Family.” *PloS One* 12 (6): e0178691.

Winsor, C. P. 1932. “The Gompertz Curve as a Growth Curve.” *Proceedings of the National Academy of Sciences of the United States of America* 18 (1): 1–8.
