# Supplementary figures and images for: Structured tracking of alcohol reinforcement (STAR) for basic and translational alcohol research
Source: Mol Psychiatry. 2023 Feb 27;28(4):1585–98. doi: 10.1038/s41380-023-01994-4 (PMC10208967; doi:10.1038/s41380-023-01994-4)

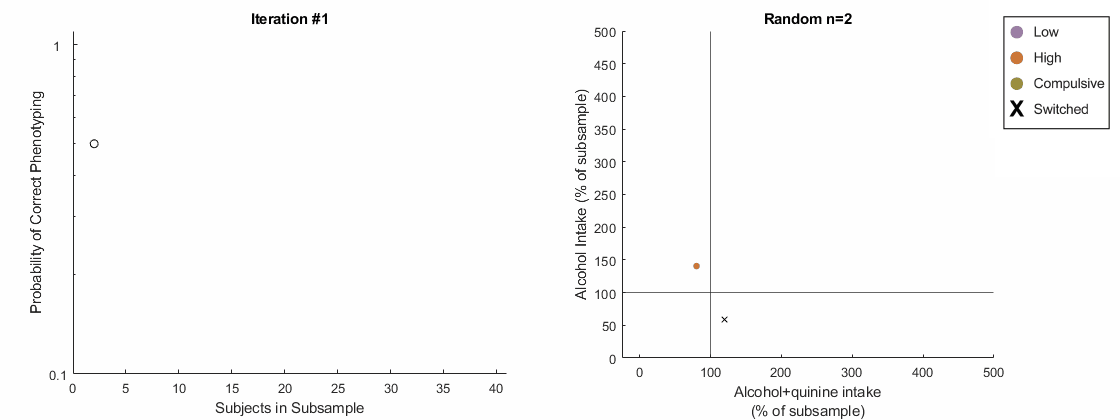

Supplement: Supplementary file 2 — Supplemental Video 1 [file 41380_2023_1994_MOESM2_ESM.gif]
