# Supplementary material for: Structured tracking of alcohol reinforcement (STAR) for basic and translational alcohol research
Source: Mol Psychiatry. 2023 Feb 27;28(4):1585–98. doi: 10.1038/s41380-023-01994-4 (PMC10208967; doi:10.1038/s41380-023-01994-4)
Supplement: Supplementary file 3 — Supplemental Video 1 Legend [file 41380_2023_1994_MOESM3_ESM.docx]

**Supplemental Video 1.** Explanatory visualization of the permutated resampling pipeline use to test sample-size dependence of STAR phenotyping. The full analysis included 100 iterations, see Supplemental Figure 10 for full methodological details.
